# PcrG protects the two long helical oligomerization domains of PcrV, by an interaction mediated by the intramolecular coiled-coil region of PcrG

**DOI:** 10.1186/1472-6807-14-5

**Published:** 2014-01-24

**Authors:** Abhishek Basu, Urmisha Das, Supratim Dey, Saumen Datta

**Affiliations:** 1 Structural Biology and Bioinformatics division, Indian Institute of Chemical Biology, 4 Raja S.C. Mullick Road, Kolkata 700032 West Bengal, India

**Keywords:** Regulation of TTSS, Functional translocon, Dynamic light scattering and elongated conformation, Homology model, Protease protected fragment, MS/MS sequence analysis, Reversal of oligomerization, Intramolecular coiled-coil, Deletion mutants, Surface plasmon resonance and protein-protein interaction, Molecular docking

## Abstract

**Background:**

PcrV is a hydrophilic translocator of type three secretion system (TTSS) and a structural component of the functional translocon. C-terminal helix of PcrV is essential for its oligomerization at the needle tip. Conformational changes within PcrV regulate the effector translocation. PcrG is a cytoplasmic regulator of TTSS and forms a high affinity complex with PcrV. C-terminal residues of PcrG control the effector secretion.

**Result:**

Both PcrV and PcrG-PcrV complex exhibit elongated conformation like their close homologs LcrV and LcrG-LcrV complex. The homology model of PcrV depicts a dumbbell shaped structure with N and C-terminal globular domains. The grip of the dumbbell is formed by two long helices (helix-7 and 12), which show high level of conservation both structurally and evolutionary. PcrG specifically protects a region of PcrV extending from helix-12 to helix-7, and encompassing the C-terminal globular domain. This fragment ∆PcrV_(128–294)_ interacts with PcrG with high affinity, comparable to the wild type interaction. Deletion of N-terminal globular domain leads to the oligomerization of PcrV, but PcrG restores the monomeric state of PcrV by forming a heterodimeric complex. The N-terminal globular domain (∆PcrV_(1–127)_) does not interact with PcrG but maintains its monomeric state. Interaction affinities of various domains of PcrV with PcrG illustrates that helix-12 is the key mediator of PcrG-PcrV interaction, supported by helix-7. Bioinformatic analysis and study with our deletion mutant ∆PcrG_(13–72)_ revealed that the first predicted intramolecular coiled-coil domain of PcrG contains the PcrV interaction site. However, 12 N-terminal amino acids of PcrG play an indirect role in PcrG-PcrV interaction, as their deletion causes 40-fold reduction in binding affinity and changes the kinetic parameters of interaction. ∆PcrG_(13–72)_ fits within the groove formed between the two globular domains of PcrV, through hydrophobic interaction.

**Conclusion:**

PcrG interacts with PcrV through its intramolecular coiled-coil region and masks the domains responsible for oligomerization of PcrV at the needle tip. Also, PcrG could restore the monomeric state of oligomeric PcrV. Therefore, PcrG prevents the premature oligomerization of PcrV and maintains its functional state within the bacterial cytoplasm, which is a pre-requisite for formation of the functional translocon.

## Background

The Gram negative bacterium *Pseudomonas aeruginosa* is an opportunistic pathogen which causes acute infections in immune-compromised individuals. It is the causative agent of nosocomial pneumonia and other infections associated with burns, wounds, urinary tract, and cystic fibrosis [1-3]. *P. aeruginosa* possesses a TTSS, which uses an injectisome for delivery of bacterial toxic effector proteins within the host cell. The injectisome comprises of a basal structure and a needle complex. At the tip of the needle a translocon is formed by a set of three translocator proteins. This structure is essential for transport of the effector proteins and regulation of TTSS [3-7]. Two of the translocators are hydrophobic (like PopB, PopD from *Pseudomonas sp.* or YopB, YopD from *Yersinia sp.*) and form pores in the host cell membrane [6-10]. While the hydrophilic translocators (like PcrV from *Pseudomonas sp.* or LcrV from *Yersinia sp.*), also known as V-antigen, form a platform for the assembly of the hydrophobic translocators [6,7,11]. The hydrophilic translocators act as protective antigens against infection and are targets for the development of vaccine [12].

PcrV is a regulator of TTSS. It is chaperoned by PcrG, which itself is a cytoplasmic regulator of TTSS. Although, PcrV is a secretory protein, PcrG is completely cytoplasmic and both of these proteins regulate the TTSS independently [13,14]. PcrV oligomerizes at the needle tip for formation of the functional translocon. The C-terminal helix of PcrV is essential for its oligomerization. For the regulation of TTSS, PcrV exists in various conformational states. It alters the structure of translocation apparatus hence, controlling secretion of the effectors [15,16]. PcrV belongs to the Ysc family of translocators, which includes LcrV, AcrV (*Aeromonas sp*.). Other important family of translocators is Inv-Mxi, which consists of IpaD, SipD and BipD proteins. Translocators belonging to Inv-Mxi family possess two distinct domains. The N-terminal domain shows similarity with the common chaperones, suggesting a self-chaperoning function of these translocators. This is contrary to the behaviour of the translocators PcrV and LcrV, which utilize their cognate chaperones PcrG and LcrG in the bacterial cytoplasm [17-20]. The crystal structure of LcrV depicts dumbbell shape with two globular domains. The grip of the dumbbell is formed by two long helices [21]. PcrG and PcrV form a 1:1 high affinity complex and the deletion of the 24 C-terminal amino acids of PcrG does not alter the affinity of the complex formation [14]. Comparatively, little is known regarding the mechanism of regulation of TTSS by PcrG, apart from the fact that it is a negative regulator of TTSS, and C-terminal residues of PcrG regulate the effector secretion. Formation of PcrG-PcrV complex is not essential for the regulation of TTSS, but it confers stability to both the proteins within the bacterial cytoplasm and prevents the misfolding of PcrV [13-15].

The existing literature mainly focuses on the function of PcrV with respect to the regulation of TTSS. In this study, we have emphasized on the structural aspects of PcrG-PcrV interaction and conclusively proposed a model for the formation of PcrG-PcrV complex.

## Results and discussion

### PcrV retains the elongated conformation in the complex with PcrG, but with structural alteration

**Figure 1:**
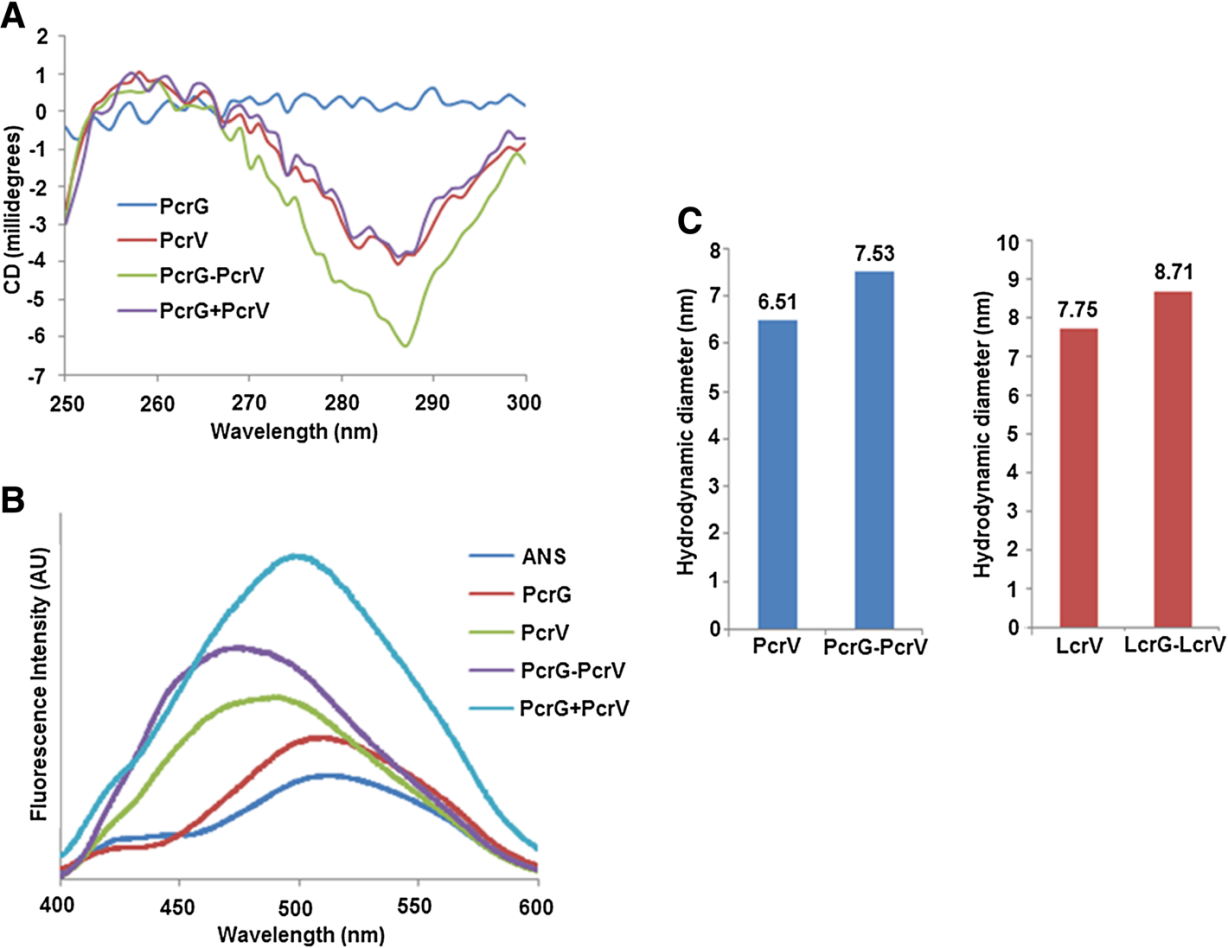
**Near UV CD, ANS-fluorescence spectra, and DLS profile show structural stabilization and elongated conformation of the proteins. A**. Near UV CD signal recorded from 300 nm to 250 nm, shows negative signal for PcrV and PcrG-PcrV at 285 nm and 287 nm, respectively. PcrG shows absence of tertiary structure signal. **B**. ANS-binding profile of proteins was scanned from 400 nm to 600 nm. Both PcrV and PcrG-PcrV exhibit a blue-shift in the ANS-binding spectra, showing the presence of solvent exposed hydrophobic patches in the proteins. However, the spectrum of PcrG was similar to that of ANS. **C**. DLS profiles of PcrV, PcrG-PcrV and LcrV, LcrG-LcrV with corresponding hydrodynamic diameters, confirm an elongated conformation of the proteins.

Based on Far UV CD spectrum and thermal denaturation curve, it was proposed that PcrV imparts structural stability to PcrG. Also, it was established that PcrG-PcrV interaction imparts stability to both the proteins [14]. The near UV CD spectrum showed the absence of tertiary structure signal for PcrG (Figure 1A). PcrV showed a negative signal at 285 nm (Figure 1A), which was almost in compliance with the previously reported minimum at 284 nm [15]. However, there was a shift of the minimum to 287 nm in case of PcrG-PcrV complex and the negative signal was more prominent in case of the complex structure (Figure 1A). This indicated towards a structural alteration of PcrV in presence of PcrG, in the complex. When PcrG was incubated with 8-anilinonapthalene-1-sulfonate (ANS), it showed almost similar spectrum to that of ANS (Figure 1B). This observation can be attributed to the lack of solvent exposed hydrophobic domains within PcrG, required for ANS binding. However, in the ANS-binding experiment, both PcrV and PcrG-PcrV complex showed significant blue shift in the λ_max_ to 484 nm and 476 nm, respectively. Moreover, the summation of PcrG-ANS and PcrV-ANS spectra was significantly different from PcrG-PcrV-ANS spectrum, revealing the differential structural moulding of both PcrG and PcrV within the complex (Figure 1B).

Dynamic light scattering (DLS) profile of PcrV and PcrG-PcrV revealed single predominant peaks corresponding to hydrodynamic diameters of 6.503 nm and 7.531 nm, respectively (Figure 1C). The estimated molecular weights of 53.1 kDa for PcrV and 74.9 kDa for PcrG-PcrV, clearly revealed their extended conformation. PcrV exists in a monomeric physiological state with a molecular weight of ~33 kDa. While PcrG-PcrV forms a 1:1 complex with a molecular weight of ~46 KDa [14]. Similar to PcrV, the elongated conformation was also observed in the crystal structure of its close homologue LcrV [21]. DLS experiments revealed that the hydrodynamic diameter of LcrV and LcrG-LcrV were 7.75 nm and 8.71 nm, respectively, in the solution state. This indicated towards the elongated conformation of LcrV in individual form and complex form (Figure 1C). PcrG and PcrV interaction is not essential for regulation of TTSS by these proteins. However, the formation of PcrG-PcrV complex provides structural stability to both the proteins and aids in proper folding and export of PcrV [13-15].

### A homology model of PcrV shows the elongated dumbbell shaped conformation

**Figure 2:**
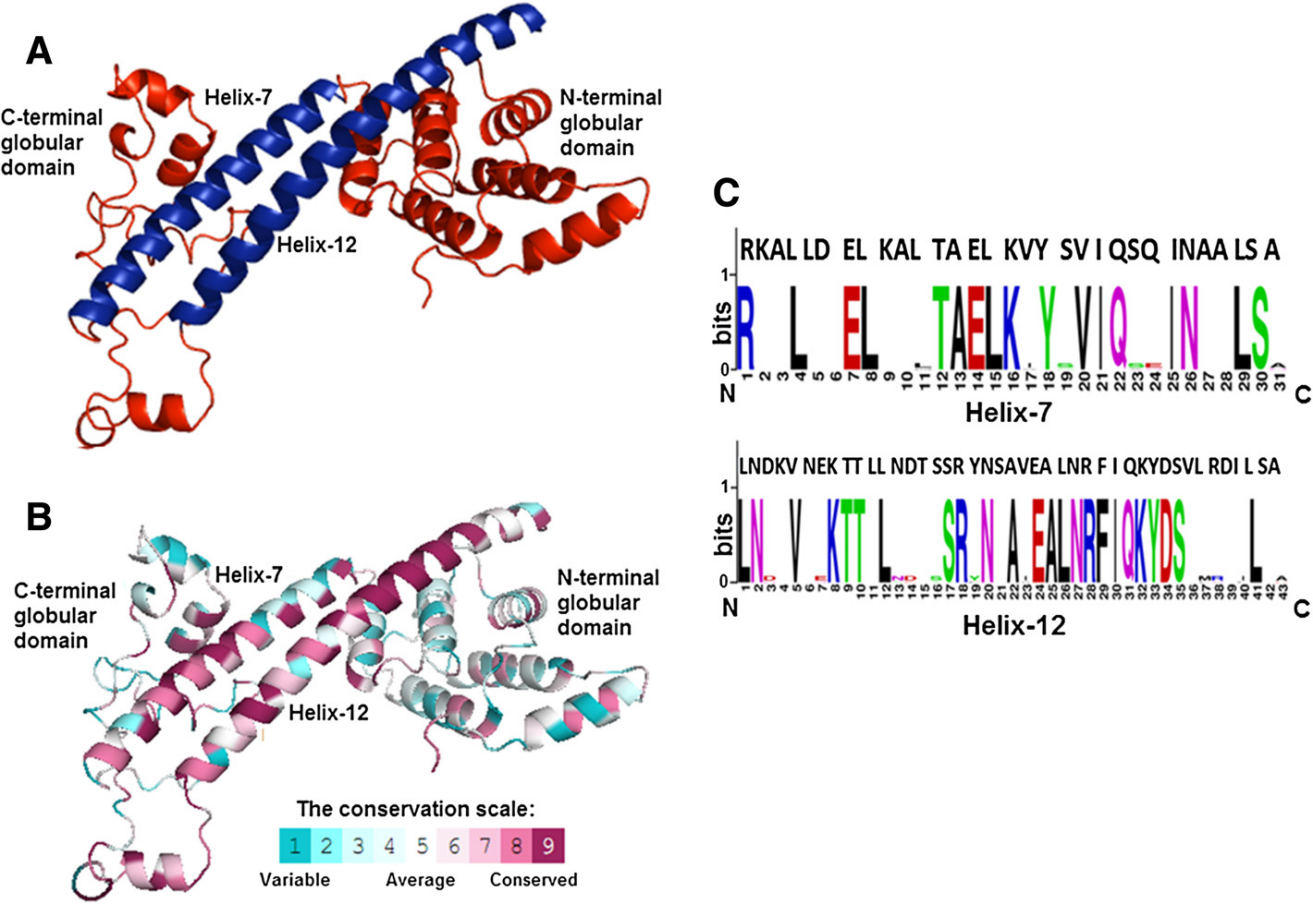
**Homology model of PcrV and its analysis indicated the structurally and functionally conserved regions. A**. Cartoon representation of homology model of PcrV depicts a dumbbell shaped structure with N and C-terminal globular domains. Helices 7 and 12 form the grip of the dumbbell. **B**. ConSurf predicted the structurally and functionally conserved residues within the homology model of PcrV, in a graded manner as shown by the colour code. **C**. WebLogo generated sequence Logos of helix-7 and 12 of V-antigens. Sequences of helix-7 and 12 of PcrV were aligned with the sequence Logos to determine the conserved positions.

The homology model of PcrV was built by I-Tasser using LcrV [PDB ID: 1R6F] as template, which has 37% sequence identity with PcrV. The homology model was validated by PROCHECK (Additional file 1, Additional file 2) [22-24]. This model has a C-score of -0.07, TM Score of 0.70 ± 0.12 and RMSD of 6.3 ± 3.8 Å. The cartoon representation of the dumbbell shaped model of PcrV depicted the predominance of α-helical structures, interspersed by coiled regions, and few β-sheets. Out of the 12 α-helices in the structure, helix-7 (128–158) and helix-12 (251–293) are the longest. These two helices run anti-parallel to each other and form the grip of the dumbbell. Two β-sheets are localized between the 2nd and 3rd, and 4th and 5th helices, respectively. There is a short helix and a long coil region extending from residues 198–233 in the C-terminal, which falls within the protective epitope region of PcrV [19]. It is also evident from the orientation of PcrV on the needle tip, that aforesaid region must be exposed to the outer environment [7,19,25]. The N-terminal globular domain is formed by α-1, α-2, β-1, α-3, α-4, β-2, α-5, α-6. This domain is predicted to interact with the needle forming protein of *P. aeruginosa*[7,25]*.* The C-terminal globular domain forming the outer part of the needle tip comprises of a short helix followed by α-8, α-9, a long coiled region and α-10, α-11. The N and C-terminal globular domains are structurally flexible due to their probable fate towards insertions (Figure 2A) [16,19,20,25]. The helix-12 is preceded by a loop in the model which might provide flexibility to the helix for attaining different conformations [15,21]. The elongated conformation proposed by the DLS data also corroborates with the dimensions of the dumbbell shaped model, which is 8.1 nm in length and 4.68 nm in width.

The homology model was used to generate ConSurf prediction model. This model is based on phylogenetic relations and evolutionary changes between homologous sequences [26]. The ConSurf model specified structurally and functionally conserved residues in a graded fashion. The helix-7, helix-12, the short helix, and the coil region preceding helix-12 showed maximum conservation. Broadly, the grip of the dumbbell is highly conserved both structurally and functionally (Figure 2B). From the multiple sequence alignment (MSA) file, a high sequence identity could be noticed between PcrV and its orthologs like AcrV, LssV and LcrV, specifically within helix-7 (residues 137–157) and helix-12 (residue 250–287) (Additional file 3) [27]. Sequence Logos of these two helices were generated using the proteins belonging to the LcrV family. The sequences of helix-7 and helix-12 of PcrV exhibited high level of conservation when compared to the consensus sequences of the Logos. In case of helix-7 (31 residues long) and helix-12 (43 residues long), 17 and 24 residues were conserved, respectively (Figure 2C) [28].

### Proteolytic digestion identified a specific region of PcrV protected by PcrG

**Figure 3:**
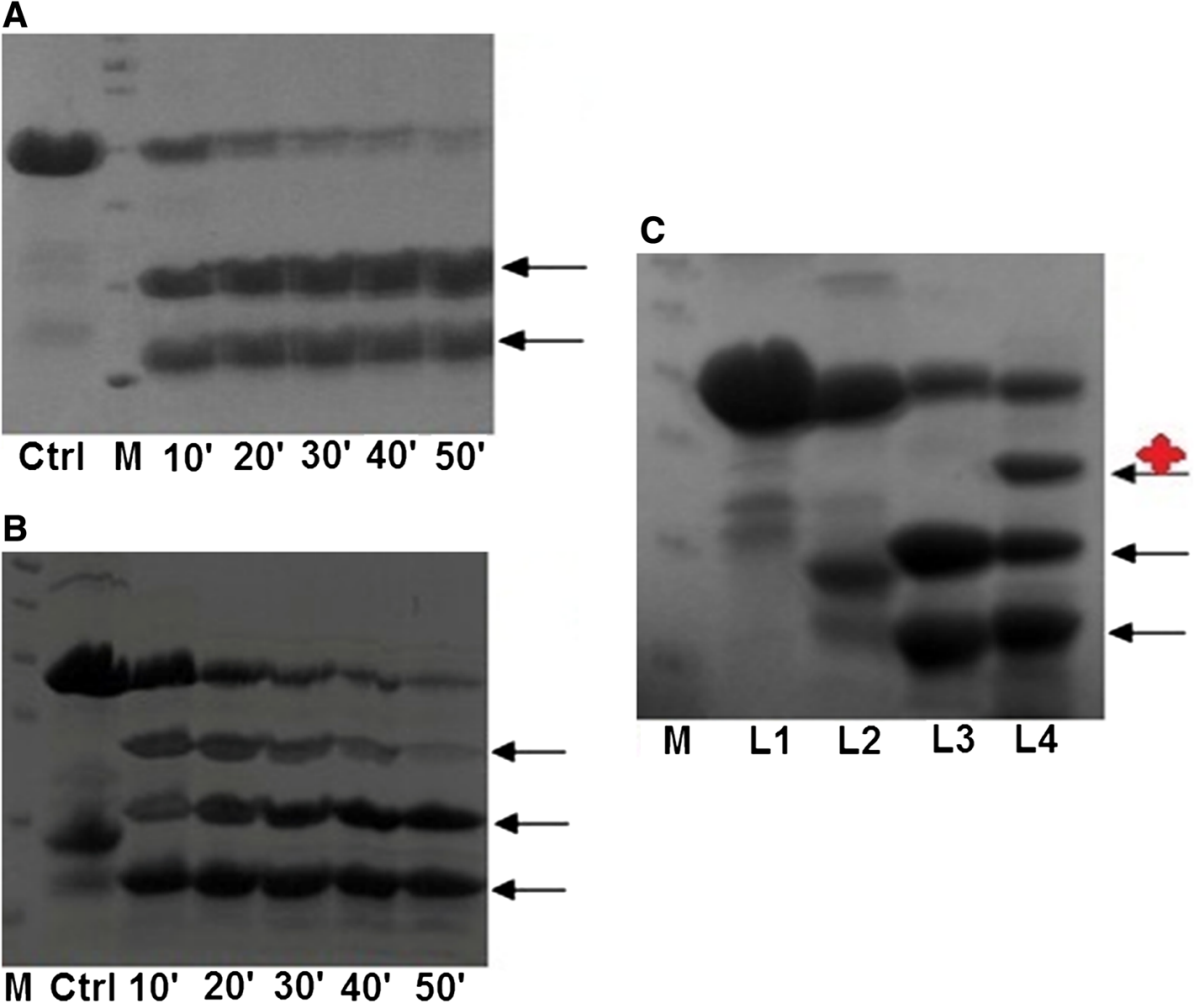
**Proteolytic digestion identified a specifically protected region of PcrV in presence of PcrG. A**. and **B**. Proteolytic digestion profiles of PcrV and PcrG-PcrV, respectively, with α-chymotrypsin from 10 to 50 minutes. Black arrows indicate the stable fragments generated after proteolysis. **C**. The digestion patterns of PcrV and PcrG-PcrV shows the presence of a specifically protected region of PcrV in the PcrG-PcrV complex, which is highlighted by a black arrow and a red star. L1 and L2 denote native PcrV and PcrG-PcrV. L3 and L4 denote PcrV and PcrG-PcrV, respectively, cleaved by α-chymotrypsin after 30 minutes. M is the protein molecular weight marker (10, 17, 26, 34, 43, 55 kDa bands from bottom to top).

Proteolytic digestion of PcrV was carried out at different time points with 1:500 dilution of α-chymotrypsin. The digestion profile showed two predominant bands (fragments) existing till 50 minutes. One band was close to the 17 kDa marker and MS/MS sequence analysis showed that this fragment approximately extended from helix-7 up to helix-12 in the C-terminal of PcrV (Figure 3A, Additional file 4). Another band was present between 10 kDa and 17 kDa. MS/MS sequence analysis revealed that this fragment consists of bulk portion of the N-terminal, and completely includes the helix-7 of PcrV (Figure 3A, Additional file 5). Similar proteolytic digestion of PcrG-PcrV (as done for PcrV) revealed the presence of an extra band in between 17 and 26 kDa, corresponding to 19487.0064 Dalton in addition to the two aforesaid bands, as observed from the mass spectrometry profile (Figure 3B & 3C, Additional file 6). So, it can be concluded that PcrG specifically protects certain interacting region of PcrV. MS/MS sequence analysis revealed that this protected region encompasses the entire C-terminal of PcrV comprising of helix-12, C-terminal globular domain and extending up to major portions of helix-7 (Additional file 7).

### PcrG restores the monomeric state of oligomeric ΔPcrV_(128–294)_ by forming a high affinity heterodimeric PcrG-ΔPcrV_(128–294)_ complex

**Figure 4:**
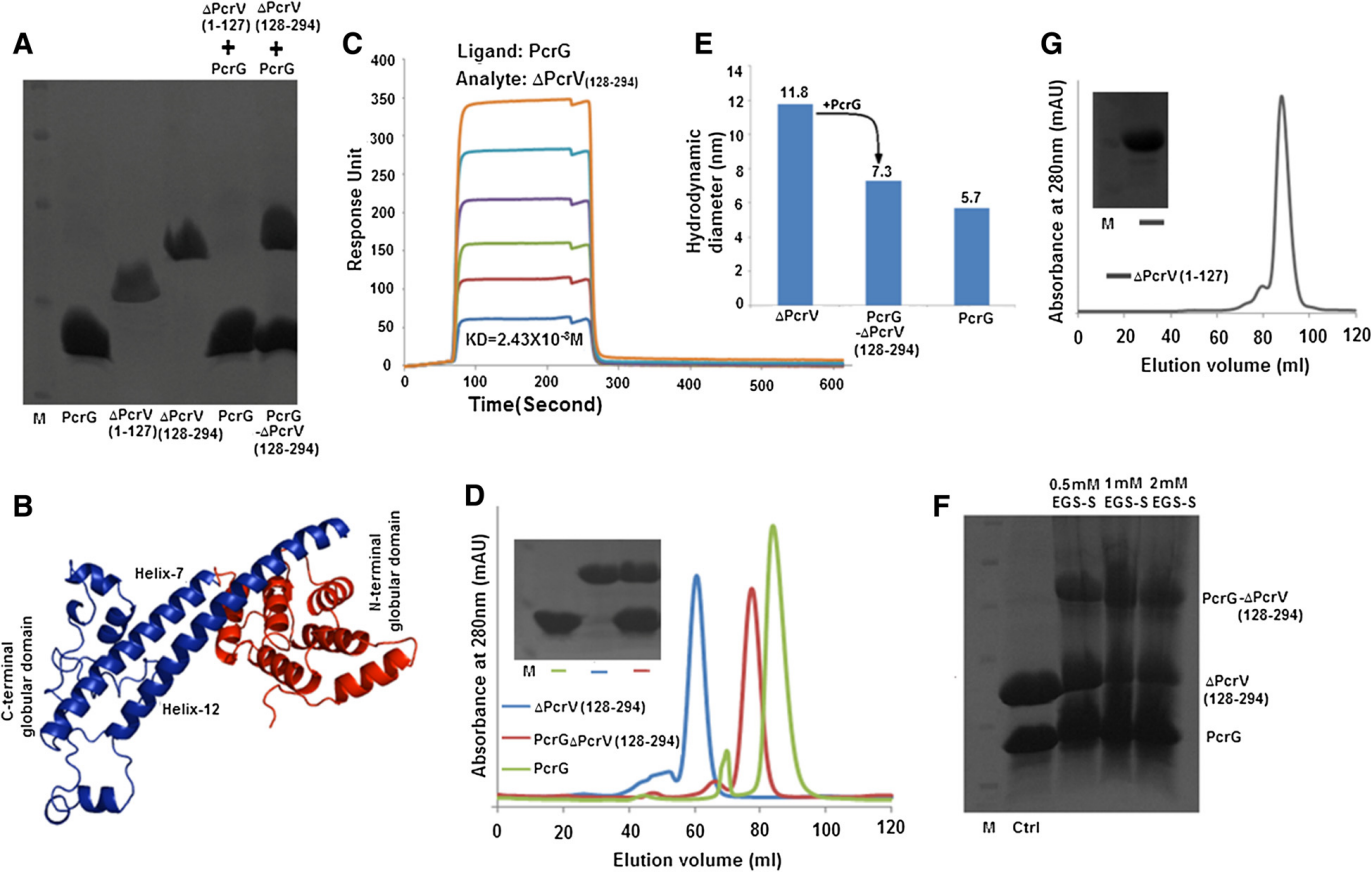
**PcrG restores the monomeric state of oligomeric ΔPcrV**_**(128–294) **_**by forming a high affinity heterodimeric PcrG-ΔPcrV**_**(128–294) **_**complex. A.** SDS PAGE showing the interaction of PcrG with ∆PcrV_(128–294)_ and ∆PcrV_(1–127)_. When ∆PcrV_(128–294)_ was incubated with PcrG and subjected to Ni-NTA affinity chromatography, both ∆PcrV_(128–294)_ and PcrG were seen in the elution fraction. However, when ∆PcrV_(1–127)_ was incubated with PcrG, only PcrG was seen in the elution. This revealed that only ∆PcrV_(128–294)_ forms a complex with PcrG. **B**. The region corresponding to ∆PcrV_(128–294)_ was shown in deep blue colour in the homology model of PcrV. This region encompasses helix-7, C-terminal globular domain, and helix-12. **C**. Surface plasmon resonance sensogram of ∆PcrV_(128–294)_ and PcrG. **D**. Size exclusion chromatography profile of ∆PcrV_(128–294)_, PcrG-∆PcrV_(128–294)_, and PcrG with the corresponding SDS PAGE showing the proteins present in each of the peaks. **E**. The DLS profile of ∆PcrV_(128–294),_ PcrG-∆PcrV_(128–294)_ complex, and PcrG with corresponding hydrodynamic diameter. **F**. ∆PcrV_(128–294)_ forms a 1:1 heterodimeric complex with PcrG, shown by chemical crosslinking with 0.5 mM, 1 mM and 2 mM EGS-sulfonate. **G**. Size exclusion chromatography profile of ∆PcrV_(1–127)_ with corresponding SDS PAGE. M denotes the protein molecular weight marker in SDS PAGE (10, 17, 26, 34, 43, 55 kDa bands from bottom to top).

Based on the results of proteolytic digestion, showing a specific region of PcrV protected by PcrG, and bioinformatic analysis showing the conservation at helix-12 and 7, we designed a deletion mutant of PcrV comprising of helix-7, the C-terminal globular domain and helix-12 (ΔPcrV_(128–294)_) (Figure 2B & 2C, Figure 3B & 3C, Additional file 6, Additional file 7). The complementary fragment of PcrV was also designed containing only the N-terminal globular domain (ΔPcrV_(1–127)_). When both ΔPcrV_(1–127)_ and ΔPcrV_(128–294)_ were incubated with PcrG and purified by affinity chromatography, the interaction studies showed that only ΔPcrV_(128–294)_ interacted with PcrG (Figure 4A). In Figure 4B, the region corresponding to ΔPcrV_(128–294)_ is highlighted in deep blue colour in the homology model of PcrV. In order to check the affinity and kinetics of interaction of ∆PcrV_(128–294)_ and PcrG, we used surface plasmon resonance (SPR). K_D_ value of 2.43 × 10^-8^ M confirmed the formation of a high affinity complex between ∆PcrV_(128–294)_ and PcrG. Although there was a slight reduction in the affinity of the complex formation (compared to PcrG-PcrV), a marked change in the association and dissociation rates of the reaction was observed (Figure 4C, Table 1) [14]. Size exclusion chromatography (SEC) profile showed that ΔPcrV_(128–294)_ exists as an oligomeric species eluting at 60 ml, which corresponds to a molecular weight ~193 kDa. We could not assign a proper state to the oligomer due to the elongated conformation of PcrV. The previous report also emphasized on the propensity of PcrV towards oligomerization on deletion of the N-terminal globular domain [20]. Gebus *et al*. [15], observed dimeric to hexameric states of PcrV on oligomerization, and concluded from further studies that V-antigens of *Y. pestis* and *P. aeruginosa* could oligomerize into higher order ring like structures with molecular weights greater than 130 kDa. However, in the complex form, PcrG-ΔPcrV_(128–294)_ showed a shift in the elution volume to 77 ml corresponding to a molecular weight of 41 kDa (actual mass of 1:1 PcrG-ΔPcrV_(128–294)_ complex is ~32 kDa), implying on the restoration of the monomeric state of ΔPcrV_(128–294)_ in the complex. PcrG elutes at 83 ml corresponding to a molecular weight of ~31 kDa, indicating towards a dimeric state (actual mass of PcrG dimer is ~25 kDa) (Figure 4D). Further, DLS was performed to corroborate the observation of SEC and to check the existence of any aggregation state of ΔPcrV_(128–294)_. DLS studies showed that ΔPcrV_(128–294)_ forms oligomeric species with a hydrodynamic diameter of 11.8 nm, suggesting a molecular weight greater than 200 kDa (Figure 4E). However, when equimolar concentration of PcrG is incubated with ΔPcrV_(128–294)_, the higher order oligomeric species attains a lower hydrodynamic diameter of 7.3 nm, which corresponds to an elongated 1:1 heterodimeric complex of PcrG-ΔPcrV_(128–294)_ (Figure 4E). This reversion of oligomeric state of ΔPcrV_(128–294)_ could be visualized by native PAGE (Additional file 8). The 1:1 heterodimeric complex could also be seen by chemically crosslinking PcrG and ΔPcrV_(128–294)_ by ethylene glycol bis [succinimidyl succinate] (EGS)-sulfonate (Figure 4 F)_._ The results depicted that the N-terminal globular domain regulates the physiological state of PcrV. The N-terminal globular domain does not interact with PcrG, but SEC profile showed that it maintains a monomeric state and it elutes at 88 ml corresponding to a ~21 kDa species (actual mass of ∆PcrV_(1–127)_ is ~15 kDa) (Figure 4G).

**Table 1:** Kinetic parameters of interaction between deletion mutants of PcrG and PcrV, were determined by SPR

**Kinetic parameter**	**PcrG-PcrV**	**∆PcrG**_**(1–74)**_**-PcrV**	**PcrG-∆PcrV**_**(128–294)**_	**∆PcrG**_**(13–72)**_**-PcrV**	**∆PcrG**_**(13–72)**_**-∆PcrV**_**(128–294)**_
**K**_**A **_**(1/M)**	6.4 × 10^7^	6.4 × 10^7^	4.11 × 10^7^	1.75 × 10^6^	1.61 × 10^6^
**K**_**D **_**(M)**	1.56 × 10^-8^	1.56 × 10^-8^	2.43 × 10^-8^	5.7 × 10^-7^	6.21 × 10^-7^
**K**_**a **_**(1/MS)**	4.45 × 10^5^	3.16 × 10^5^	5.11 × 10^2^	1.71 × 10^4^	2.72 × 10^1^
**K**_**d **_**(1/S)**	6.94 × 10^-3^	4.91 × 10^-3^	1.24 × 10^-5^	9.78 × 10^-3^	1.69 × 10^-5^

Table 1 Binding affinities, and association and dissociation rates of interaction between deletion mutants of PcrG and PcrV, were determined by SPR. Kinetic parameters of PcrG-PcrV interaction and ∆PcrG_(1–74)_-PcrV interaction were adapted from Nanao *et al.*[14].

The high affinity interaction between ∆PcrV_(128–294)_ and PcrG indicates that the location of PcrG-binding site could be within the two long helices of PcrV. Oligomerization of PcrV subunits at the tip of the needle is proposed to occur by intramolecular exchange of helix-7 and helix-12 by “domain swapping” mechanism and the last 41 amino acids forming helix-12 is essential for oligomerization. Deletion of the C-terminal helix inhibits the multimerization of PcrV and has post-secretory implications, abolishing the bacterial cytotoxicity [15]. So, protection or masking of these helices by PcrG, prevented the oligomerization and misfolding of PcrV within the bacterial cytoplasm. It also helped to maintain the functional form of PcrV by formation of the heterodimeric complex of PcrG-PcrV in the cytoplasm. The result established the neutral role of the N-terminal domain of PcrV in interaction with PcrG. Therefore, the N-terminal is responsible for the maintenance of the non-oligomeric state of PcrV and regulates the secretion of PcrV through the injectisome. Specifically, deletion of 3–20 amino acids leads to defects in the secretion of the PcrV [13]. Furthermore, the N-terminal globular domain of PcrV interacts with the needle forming protein PscF. Also, this domain of LcrV is essential for recruitment of YopB in target cell membrane. These observations prompted us to propose a chaperoning activity associated with this domain, as seen in Inv-Mxi family of hydrophilic translocators like IpaD, SipD, and BipD [3,7,19,25].

### Helix-12 is the key mediator for PcrG-PcrV interaction and helix-7 might support the interaction

From the proteolytic cleavage data, it was established that PcrG protects the C-terminal helix-12 of PcrV and major part of helix-7 (Figure 3B & 3C, Additional file 6, Additional file 7). Lee *et al*. [13], reported that mutations in C-terminal helix of PcrV affect PcrG-PcrV interaction. The F279R mutation in helix-12 has a deleterious effect towards PcrG interaction and L262D mutation abolished PcrG binding. However, the entire region of PcrV involved in PcrG-interaction is still unknown. To specifically assign the PcrG-interaction domain of PcrV, we have dissected PcrV into various fragments, comprising of a combination of four domains (i.e. N-terminal globular domain, helix-7, C-terminal globular domain, and helix-12).

**Figure 5:**
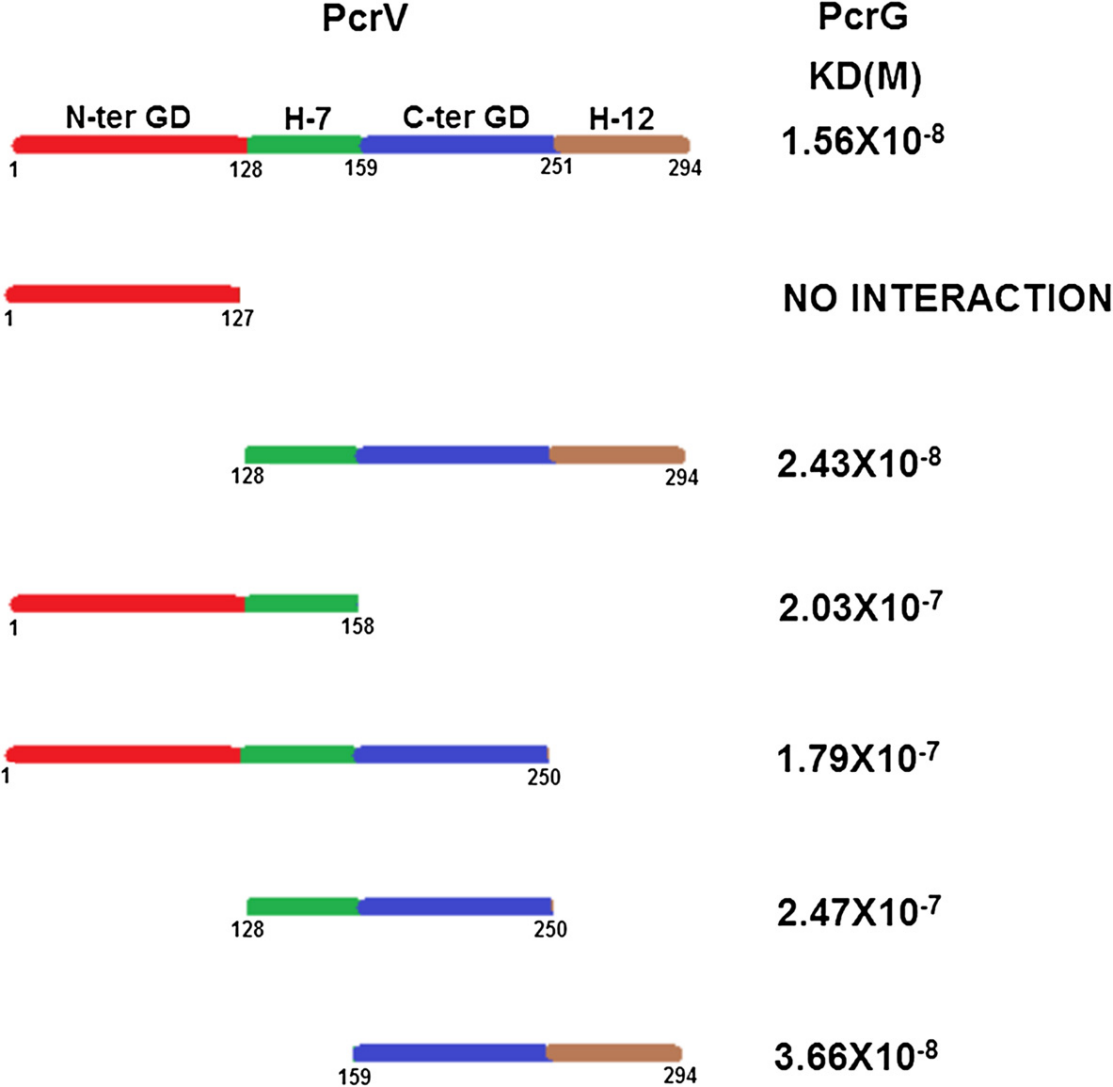
**Interaction of various domains of PcrV with PcrG.** K_D_ values estimated by SPR, reveal the binding affinities of various deletion mutants of PcrV with PcrG. Higher affinity of interaction was observed when helix-12 is present in the deletion mutant, but presence of helix-7 also allows the interaction.

Apart from the stable fragment of PcrV protected by PcrG, which is ΔPcrV_(128–294)_, we designed four more deletion fragments of PcrV to understand the role of various domains of PcrV in PcrG-PcrV interaction. ΔPcrV_(1–158)_ comprises of the N-terminal globular domain and helix-7; ΔPcrV_(1–250)_ contains the N-terminal globular domain, helix-7 and the C-terminal globular domain; ΔPcrV_(128–250)_ comprises of helix-7 and the C-terminal globular domain, and ΔPcrV_(159–294)_ contains the C-terminal globular domain and helix-12. Molecular masses of all these deletion mutants were checked by native mass spectrometry, and their experimental masses were extremely close to their theoretical masses. Quantitative binding of PcrG with each of the deletion mutants of PcrV, was analyzed by SPR. We have found that PcrG-ΔPcrV_(159–294)_ has K_D_ of 3.66 × 10^-8^ M. So, we observed a 1.5 fold reduction in the binding affinity on deletion of helix-7, compared to K_D_ of PcrG-ΔPcrV_(128–294)_ complex, however, the order of interaction remains 10^-8^ M (Figure 5). Interactions of PcrG with fragments of PcrV devoid of helix-12- ΔPcrV_(1–158)_, ΔPcrV_(1–250)_, ΔPcrV_(128–250)_ showed K_D_ values 2.03 × 10^-7^ M, 1.79 × 10^-7^ M and 2.47 × 10^-7^ M, respectively (Figure 5). These results emphasized that deletion of helix-12 leads to greater than 10 fold reduction in binding affinity, when the binding affinities of ΔPcrV_(128–294)_ and ΔPcrV_(128–250)_ were compared (Figure 5). By comparing the binding affinity of ΔPcrV_(1–158)_ and ΔPcrV_(1–250)_, we conclude an insignificant role of the C-terminal globular domain in PcrG-PcrV interaction (Figure 5).

The above experimental evidences suggest that the main PcrG-interaction domain is localized within helix-12. However, helix-7 also enables PcrG-PcrV complex formation, so it might stabilize the PcrG-PcrV interaction due to the close packing of the helices, as observed in the model. Lawton *et al.*[29], identified certain residues within helix-7 of LcrV, involved in LcrG-LcrV interaction. These residues occupy crucial positions of the heptad repeats in helix-7; hence, they are critical for the formation of intramolecular coiled-coil and α7-α12 interaction. Mutagenesis of these residues affects the binding between LcrV and LcrG [21,29,30]. Insertional and point mutagenesis identified residues in helix-12 crucial for LcrG-LcrV interaction [30]. Therefore, in LcrV, helix-7 as well as helix-12 possesses residues involved in LcrG-LcrV interaction. Interestingly, mutation of the residues in α7 of LcrV (involved in LcrG interaction) affects multimerization of LcrV, which might be due to disruption of intramolecular coiled-coil and α7-α12 interaction, but it allows the formation of the complex between LcrG-LcrV [29]. Mutations in C-terminal helix-12 of PcrV perturb the formation of the intramolecular coiled-coil and abolish the oligomerization of PcrV, revealing that formation of the coiled-coil is essential for oligomerization of the V-antigen [15]. Also, PcrG could prevent the oligomerization of PcrV, giving an indication that PcrG binding might affect the intramolecular coiled-coil structure of PcrV. Based on the crystal structure of LcrV and mechanism of LcrG-LcrV interaction mediated through the heptad repeats of α7 of LcrV, Gebus *et al*. [15], proposed that V-antigen could exist in a closed and an open state. The closed state corresponds to the monomeric shape of the V-antigen, while the open state renders α7 and α12 free for interaction with its partners [15,21,29]. Therefore, the formation of intramolecular coiled-coil is not absolutely essential for the interaction of V-antigen with its partners. These facts corroborate our SPR analysis, where we have observed that the presence of either of the two helices (7 or 12) in PcrV enables the complex formation with PcrG, because both the helices contain residues for PcrG interaction. Structural stabilization by intramolecular coiled-coil structure is not an absolute necessity for PcrG-PcrV interaction. However, the affinity of the interaction is reduced due to the changes in the local structure in absence of one of the helices. Single mutation of L262D in helix-12 of PcrV, abolishes PcrG-PcrV interaction [13]. The corresponding residue L291 in LcrV is not only involved in LcrG interaction, but also present within the zipper motif and takes part in α7-α12 interaction [30]. Importantly, L262D mutation inhibits the oligomerization of PcrV and had a profound effect on bacterial cytotoxicity [13,15]. Therefore, under the influence of this mutation V-antigen attains a monomeric shape corresponding to its “closed conformation”, which is not favourable for the interaction of V-antigen with its binding partners [15]. Changing the hydrophobic leucine to charged aspartic acid residue might enforce a local structural change within both the helices due to their close packing. So, it would be interesting to test whether other single amino acid substitution in the helix-12 leading to oligomerization defects, could also abolish PcrG-PcrV interaction.

### Intramolecular coiled-coil region of PcrG contains the PcrV interaction site with an indirect role of N-terminal residues of PcrG in the interaction

**Figure 6:**
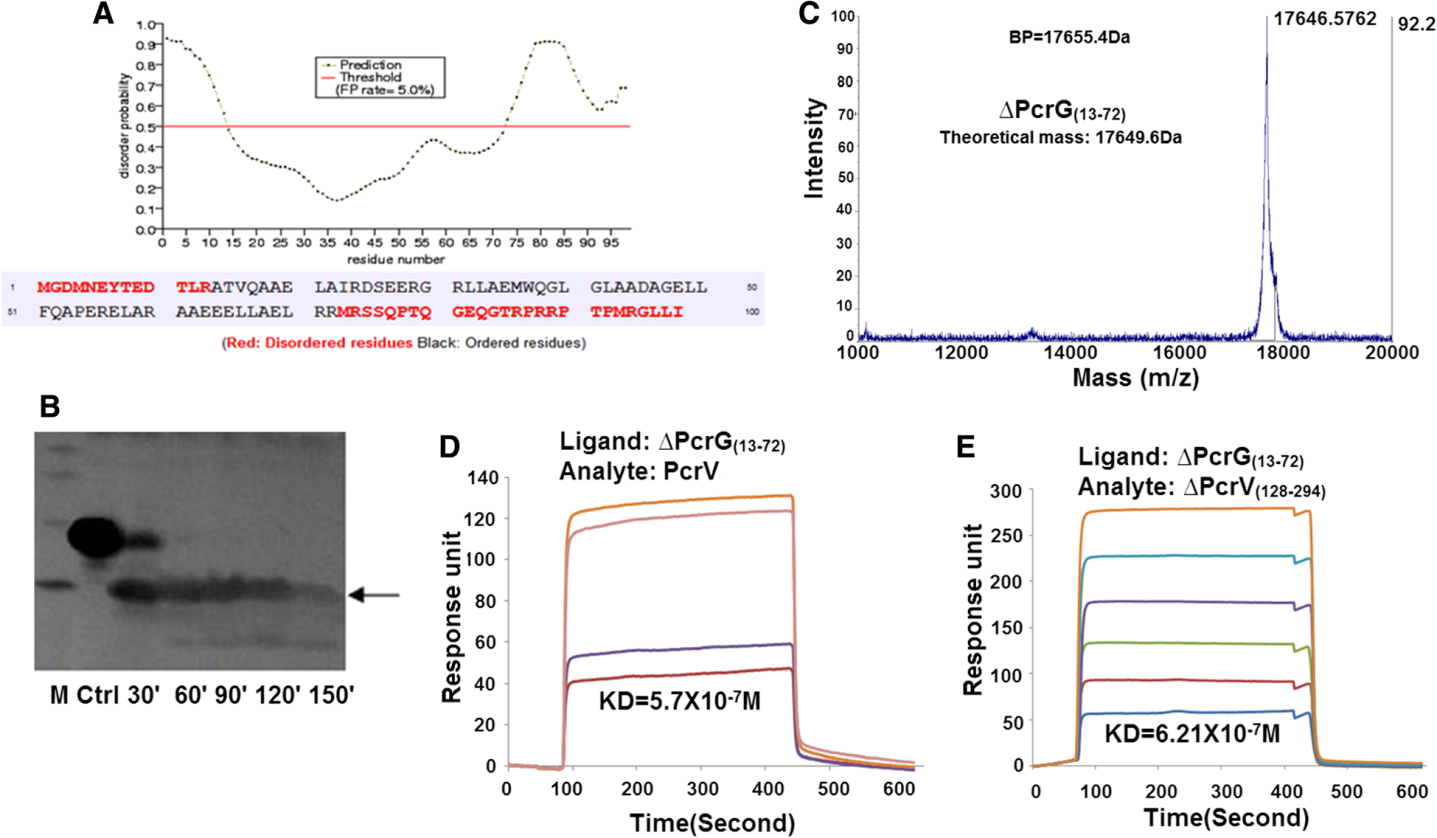
**∆PcrG**_**(13–72) **_**containing first intramolecular coiled-coil region of PcrG shows interaction with PcrV and ∆PcrV**_**(128–294)**_**. A**. Disordered regions in PcrG predicted by PrDOS. The image was directly taken from the server. **B**. Proteolytic digestion of PcrG with elastase from 30 to 150 minutes. Ctrl denotes native PcrG and M is the protein molecular weight marker (10, 17, 26, 34 kDa bands from bottom to top). **C**. Native mass spectrometry shows the dimeric state of ∆PcrG_(13–72)_. **D**. Surface plasmon resonance sensogram of ∆PcrG_(13–72)_ and PcrV shows a 40 fold reduction in affinity of interaction on deletion of the 12 N-terminal amino acids of PcrG. **E**. Surface plasmon resonance sensogram of ∆PcrG_(13–72)_ and ∆PcrV_(128–294)_.

24 C-terminal amino acids of PcrG, exhibit disorder and absence of secondary structure [14]. Further analysis of PcrG with various disorder prediction servers revealed that a few amino acids in the N-terminal and a patch in the C-terminal are disordered. PrDOS predicted disorder region of 1–13 amino acids in the N-terminal and 73–98 in the C-terminal (Figure 6A) [31]. DisEMBL 1.5 and Disopred version-2.0 provided almost similar predictions (Additional file 9) [32,33]. Controlled proteolytic digestion with elastase produced a fragment of PcrG with molecular weight of 9782.1182 Dalton, with an intact C-terminal, as revealed by mass spectrometry and MS/MS sequence analysis (Figure 6B, Additional file 10, Additional file 11). The C-terminal region is an essential part of PcrG since, deletion of this region leads to deregulation of effector secretion [13]. Also, MSA showed that there is substantial homology and conservation in the C-terminal of PcrG (Additional file 12) [27]. Though the C-terminal of PcrG is disordered, it is inaccessible to ANS and protected from proteolytic digestion. This may be due to the formation of compact structure.

When PcrG was divided into two fragments one comprising of amino acid 2–40 and the other consisting of amino acid 41–95, only the former fragment interacted with PcrV [13]. Also, PcrG and its homologs like AcrG, LssG and LcrG show maximum identity and conservation between residues 20–35 (Additional file 12) [27]. The COILS/PCOILS server predicted that amino acids 9–31 constitute the first intramolecular coiled-coil region in PcrG (Additional file 13) [34]. The N-terminal intramolecular coiled-coil region is essential in case of LcrG for its interaction with LcrV [29,35]. Based on the above results, we have designed a deletion mutant of PcrG, comprising of amino acids 13–72. This deletion mutant (∆PcrG_(13–72)_) interacts with PcrV, undermining the role of first 12 N-terminal residues of PcrG in PcrG-PcrV interaction. Similar to PcrG, ∆PcrG_(13–72)_ also exists in a dimeric state, as detected by native mass spectrometry. The theoretical mass of ∆PcrG_(13–72)_ dimer is 17649.6 Da and the observed experimental mass is 17646.5762 Da (BP = 17655.4 Da) (Figure 6C). However, due to the deletion of the predicted disordered region, the helicity of ∆PcrG_(13–72)_ increases to ~24% (data to be published).

Deletion of 24 C-terminal residues of PcrG does not have any effect on the affinity of PcrG-PcrV interaction [14]. After deletion of the first 12 N-terminal amino acids and the 26 C-terminal amino acids of PcrG, ∆PcrG_(13–72)_ could still form a high affinity complex with PcrV (K_D_ 5.7 × 10^-7^ M). But there are certain alterations in the association and dissociation kinetics (Figure 6D, Table 1) [14]. Since, amino acid 2–40 of PcrG interacts with PcrV [13] and our deletion mutant of PcrG containing residue 13–72, also interacts with PcrV, we predict that residues 13–40 of PcrG form the core region for PcrG-PcrV interaction. Moreover, this region contains almost the entire intramolecular coiled-coil and amino acids A19, S26 and L33. These three amino acids are conserved and are key residues involved in LcrG-LcrV interaction [34,35]. Although the deletion of 12 N-terminal residues abolishes LcrG-LcrV interaction [35], similar deletion of 12 N-terminal residues in PcrG could still allow it to form a high affinity complex with PcrV. However, a 40-fold reduction in binding affinity was noticed when the K_D_ value of PcrG-PcrV and ∆PcrG_(13–72)_-PcrV were compared (Figure 6D, Table 1) [14]. ∆PcrG_(13–72)_ also interacts with ∆PcrV_(128–294)_ with a slight reduction in the affinity (K_D_ 6.21 × 10^-7^ M), compared to full length PcrV (Figure 6E, Table 1). It may predict an indirect involvement of the N-terminal in the interaction between PcrG and PcrV. The structural domains of PcrG involved in PcrG-PcrV interaction, might differ from those of LcrG in LcrG-LcrV interaction.

**Figure 7:**
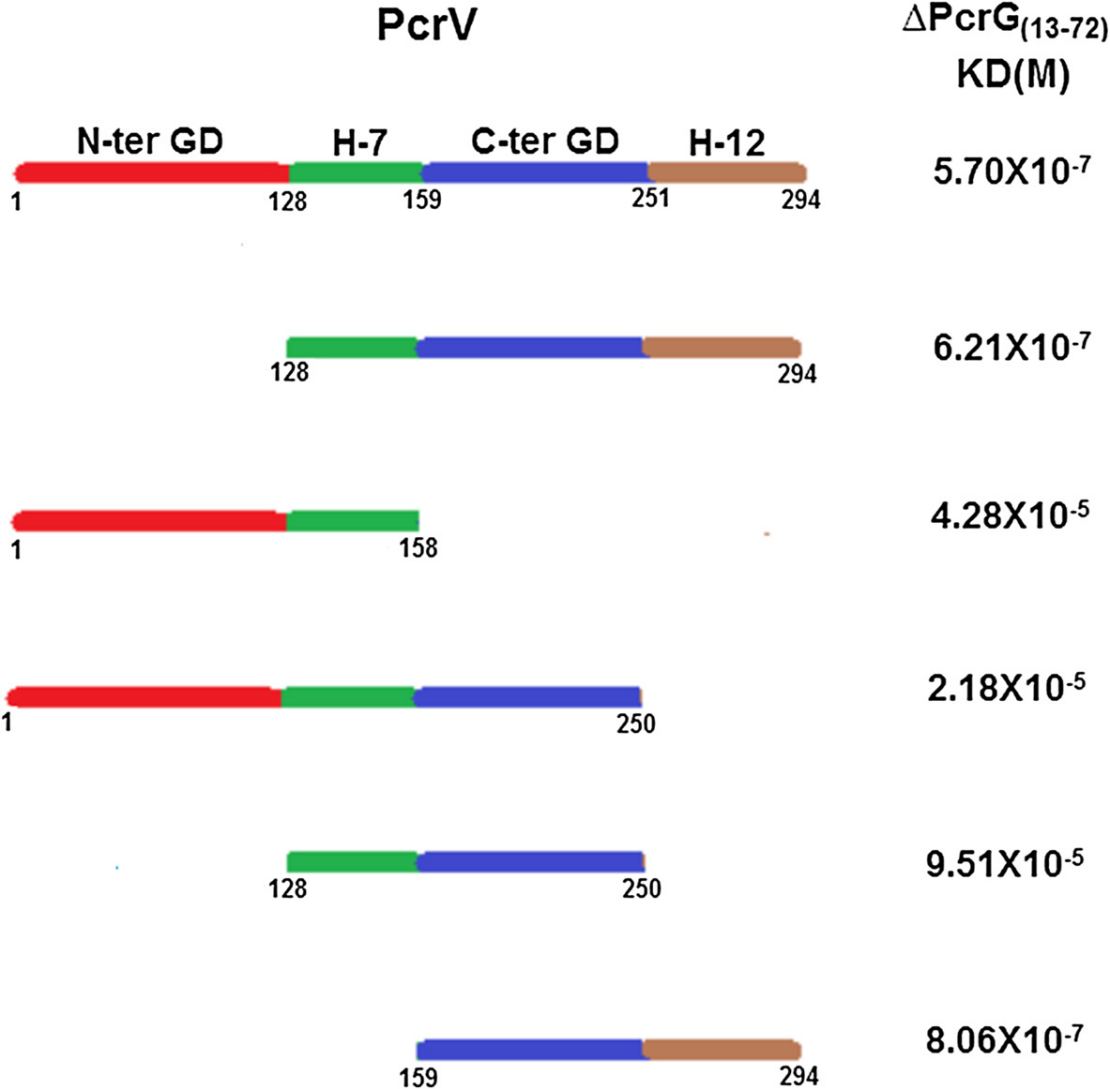
**Interaction of various domains of PcrV with ∆PcrG**_**(13–72)**_**.** K_D_ values estimated by SPR, show the binding affinities of various deletion fragments of PcrV with ∆PcrG_(13–72)_.

During the interaction of ΔPcrG_(13–72)_ with ΔPcrV_(1–158)_, ΔPcrV_(1–250)_, and ΔPcrV_(128–250)_, the K_D_ reduces to micromolar range. This high reduction in the binding affinity could be attributed to the absence of helix-12 of PcrV and 12 N-terminal residues of PcrG. But ΔPcrG_(13–72)_ and ΔPcrV_(159–294)_ (fragment containing helix-12) still show nanomolar range of interaction, emphasizing the significant role of helix-12 in the PcrG-PcrV interaction (Figure 7). In spite of deletion of various domains of PcrV and PcrG, presence of either of the helices (7 or 12) in PcrV and intramolecular coiled region of PcrG enables the formation of the complex. These observations further established that helix-7 or 12 of PcrV and intramolecular coiled-coil of PcrG contain the sites for PcrG-PcrV interaction.

### Finally a model of ∆PcrG_(13–72)_ and docking studies put ∆PcrG_(13–72)_ in a groove formed between two globular domains of PcrV

**Figure 8:**
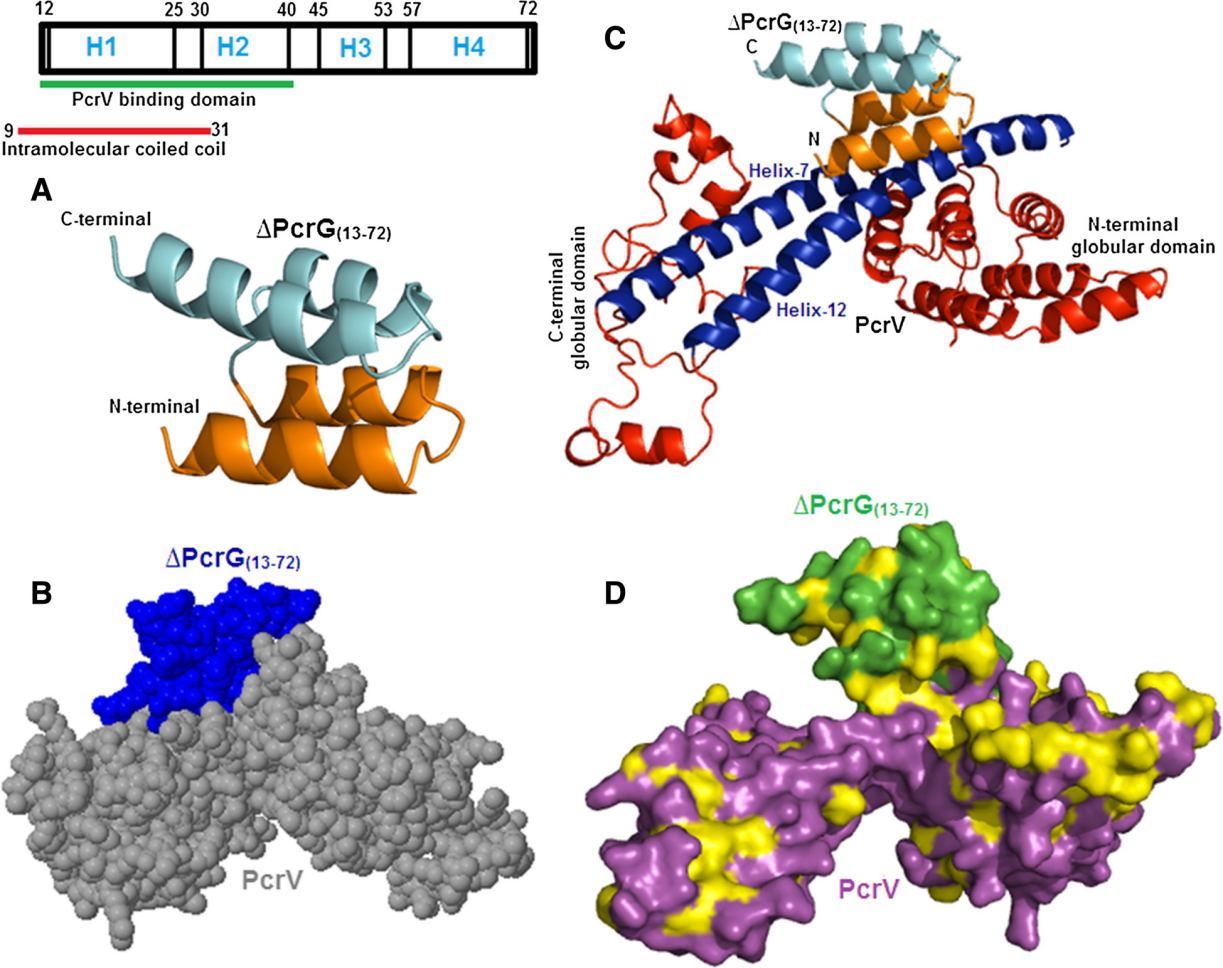
**Molecular docking fits ∆PcrG**_**(13–72) **_**into the groove formed between the two globular domains of PcrV. A**. Schematic and Cartoon representation of ∆PcrG_(13–72)_ model, with the first two helices shown in orange colour. **B**. Spacefill model of PcrG-PcrV complex obtained from molecular docking studies (∆PcrG_(13–72)_ was shown in blue and PcrV in grey colour), depicts that ∆PcrG_(13–72)_ localizes within a groove formed between the two globular domains of PcrV. **C**. Cartoon representation of model of ∆PcrG_(13–72)_-PcrV complex. The interacting regions of the ∆PcrG_(13–72)_ was shown in orange and that of PcrV was shown in blue, as represented in their respective models. **D**. Surface representation of ∆PcrG_(13–72)_-PcrV complex, where ∆PcrG_(13–72)_ and PcrV were shown in green and magenta, respectively. The hydrophobic amino acids were coloured in yellow. This model reveals that the interface of interaction between ∆PcrG_(13–72)_ and PcrV is mainly hydrophobic.

A model of the deletion mutant of PcrG (∆PcrG_(13–72)_) was generated using I-Tasser (Additional file 14). Since, experimental 3D structures of orthologs of PcrG were unavailable; I-tasser used a threading algorithm. Deletion of the disordered regions of PcrG led to a significant improvement in the quality of the model. C-score and the TM Score of ∆PcrG_(13–72)_ model are -1.5 and 0.52 ± 0.12, respectively, which satisfied the I-Tasser cut off for a correct model, and confirmed a similar topology between the model and the templates. The model was further validated by PROCHECK (Additional file 15) [22-24]. However, template proteins selected by threading programmes of I-Tasser were not orthologous to the modelled protein, and the process is based on prediction of similar fold between the proteins. If not the actual state of PcrG, the model provides the basic scaffold structure to estimate the binding site of PcrV, which was already verified by experimental techniques. The model of ∆PcrG_(13–72)_ represents four helices interspersed by coiled regions. The predicted residues of PcrG involved in PcrV-binding, exactly map to the first two helices of the model (shown in orange colour) [Figure 4A]. Molecular docking studies were performed using ZDOCK version ZD 3.0.2, where PcrV was designated as the receptor and ∆PcrG_(13–72)_ as the corresponding ligand. The best model obtained from docking studies was selected and validated by PROCHECK [24,36] (Additional file 16, Additional file 17). The spacefill model of ∆PcrG_(13–72)_-PcrV complex depicted that the binding pocket of ∆PcrG_(13–72)_ was located within the groove (or grip), in between the two globular terminal domains of PcrV (Figure 8B). The cartoon representation showed that the first two helices of ∆PcrG_(13–72)_, which overlap with the first predicted intramolecular coiled-coil domain, specifically interacts with helix-7 and helix-12 of PcrV (Figure 8C). These observations further confirmed the fact that 13–40 residues of PcrG and two long helices of PcrV are the key mediators for the formation of the complex. Figure 8D depicted the surface representation of ∆PcrG_(13–72)_-PcrV complex, where the hydrophobic residues were coloured in yellow. This model shows that ∆PcrG_(13–72)_ and PcrV interact through a hydrophobic interface corroborating the concept of hydrophobic residues being involved in interaction of V-antigen with its regulator [13,29,30,35].

## Conclusions

PcrG and PcrV form a high affinity complex, which confers stability and maintain the functional physiological states of both the proteins within the bacterial cytoplasm [13,14]. Like its homolog LcrV, PcrV exhibits an extended conformation, both in individual and in complex form. PcrG protects a specific region of PcrV extending from helix-7 to helix-12, encompassing the C-terminal globular domain, and devoid of the N-terminal globular domain. However, the deletion of the N-terminal globular domain of PcrV leads to its oligomerization, which could be reverted back by PcrG. Therefore, PcrG can restore the monomeric form of oligomer formed by PcrV. It was seen that helix-12 of PcrV plays the key role in PcrG-PcrV interaction, supported by helix-7. N and C-terminal globular domains have neutral roles in the interaction. However, the N-terminal globular domain of V-antigen maintains a monomeric state. It interacts with needle forming protein PscF and recruits hydrophobic translocator in the target cell membrane, thereby, indicating towards a chaperoning function [7,19,25].

Interestingly, formation of the functional translocon capable of targeting effectors within the host cell requires oligomerization of PcrV at the needle tip [3-7,11,15,25]. Since, the PcrG interaction domain overlaps with the oligomerization domain of PcrV, PcrG could potentially stop the oligomerization of PcrV by docking into the groove formed by helix-7 and helix-12. The intramolecular coiled-coil region of PcrG contains the PcrV binding domain, but unlike LcrG, the 12 N-terminal residues of PcrG only have an indirect role in PcrG-PcrV interaction. To conclude, PcrG prevents the premature oligomerization of PcrV and maintains its functional state within the bacterial cytoplasm, which is a pre-requisite for the formation of functional translocon.

## Methods

### Design of expression vectors

The genes of *pcrG, pcrV* and *pcrG-pcrV, ∆pcrG*_*(13–72)*_*, ∆pcrVs* (deletion constructs of *pcrV*) were amplified by PCR, using the chromosomal DNA of *P. aeruginosa* strain 2192. *pcrG* was cloned with EcoRI and HindIII, using sense primer TA**GAATTC**TATGGGCGACATGAACGAA and antisense primer AAT**AAGCTT**TCAGATCAACAAGCCACG in pETDuet-1. *∆pcrG*_*(13–72)*_ was cloned in pET-28a (+) with NdeI and XhoI, using sense primer TTAGGATC**CATATG**CGGGCGACCGTCCAGGCC and antisense primer TTA**CTCGAG**TTAGCGCCGCAGTTCGGCCAG. *pcrV, ∆pcrV*_*(1–127)*_*, ∆pcrV*_*(1–158),*_ and *∆pcrV*_*(1–250)*_ were cloned in pET-22b-∆50CPD with NdeI and EcoRI, using sense primer AATCCATGGAA**CATATG**GAAGTCAGAAACCT and antisense primers TAA**GAATTC**GGGATCGCGCTGAGAATGTCGC, TAA**GAATTC**GGCTTGCCGTCCTGGGTCTG, TAA**GAATTC**GGCTTGGCCGACAGCGCGGC, and TAA**GAATTC**GGCGGACGCGAGCGGTCGCT, respectively. *∆pcrV*_*(128–294)*_ and *∆pcrV*_*(128–250)*_ were cloned in pET-22b-∆50CPD with NdeI and EcoRI, using sense primer AATCCATGGAA**CATATG**CGCAAGGCGCTGCTCGAC and antisense primers TAA**GAATTC**GGGATCGCGCTGAGAATGTCGC and TAA**GAATTC**GGCGGACGCGAGCGGTCGCT, respectively. *∆pcrV*_*(159–294)*_ was cloned in pET-28a (+) with NdeI and HindIII, using sense primer AATCCATGGAA**CATATG**CAGGGCATCAGGATCGAC and antisense primer TTA**AAGCTT**CTAGATCGCGCTGAGAAT. *pcrG-pcrV* was cloned in pETDuet-1 with EcoRI and HindIII, using sense primer of *pcrG* TA**GAATTC**TATGGGCGACATGAACGAA and antisense primer of *pcrV* TTA**AAGCTT**CTAGATCGCGCTGAGAAT. The restriction sites were marked in bold letters. *E. coli* Top10 was used as the cloning strain.

### Purification of proteins

The proteins were expressed in BL21 DE3 by culturing in Luria Bertani medium and induction was carried with 1 mM IPTG for 4 hours at 37°C. Only ∆PcrG_(13–72)_ was induced overnight at 22°C. 1 mM phenylmethanesulfonyl fluoride was used as protease inhibitor. PcrG, ∆PcrG_(13–72)_ and PcrG-PcrV have N-terminal 6X histidine tag. These proteins were purified using standard nickel-nitrilotriacetic acid (Ni-NTA) affinity chromatography. A step gradient of immidazole was applied in 25 mM Tris–HCl (pH-8) and 150 mM NaCl buffer, for removing the non-specific proteins and eluting the desired protein. PcrV and its deletion mutants (∆PcrV_(1–127)_, ∆PcrV_(1–158)_, ∆PcrV_(1–250)_, ∆PcrV_(128–250)_, and ∆PcrV_(128–294)_) were fused to a cysteine protease domain (CPD) at their C-terminal. This CPD possesses a C-terminal histidine tag. The fusion product of the target protein and CPD is engineered in such a manner that inositol hexokisphosphate recognizes a specific leucine residue and cleaves exactly at the junction of the PcrV and the CPD. PcrV-CPD was immobilized in Ni-NTA affinity column and treated with 500 μM inositol hexokisphosphate in 25 mM Tris–HCl (pH-7.5) and 150 mM NaCl buffer. Inositol hexokisphosphate cleaves at the junction of PcrV and CPD to release PcrV (without histidine tag) from the affinity column, and CPD with the C-terminal histidine tag remains attached with the Ni-NTA. The deletion mutants of PcrV were purified in a similar way. So, we obtain PcrV and its various forms without a histidine tag for further downstream experiments. For detailed procedure refer Shen *et al.*[37]. ∆PcrV_(159–294)_ had localized in the inclusion bodies. The inclusion bodies were denatured using 6 M guanidium hydrochloride. ∆PcrV_(159–294)_ was refolded by slow dialysis in 25 mM Tris–HCl (pH-8), 150 mM NaCl and 10% glycerol buffer and subjected to Ni-NTA chromatography. Further the histidine tag of ∆PcrV_(159–294)_ was removed by thrombin digestion which cleaves the leader sequence. All the proteins were checked for their purity and molecular weight by native mass spectrometry. For purification of PcrG-∆PcrV_(128–294)_, PcrG was immobilized in the Ni-NTA column and incubated with ∆PcrV_(128–294)_ (without histidine tag). The column was washed thoroughly to remove any unbound protein and protein was eluted by a step gradient of immidazole in 25 mM Tris–HCl (pH-8) and 150 mM NaCl buffer. Both the proteins were observed in the elution fraction. Similar incubation of PcrG with ∆PcrV_(1–127)_ and subsequent elution from the affinity column showed the presence of ∆PcrV_(1–127)_ in wash fraction and PcrG in elution fraction, revealing that ∆PcrV_(1–127)_ is not forming a complex with PcrG. Purified proteins were dialyzed to remove immidazole or 500 μM inositol hexokisphosphate, in buffer according to the need of the downstream experiments.

### Size exclusion chromatography

Proteins purified by Ni-NTA chromatography were dialyzed in 25 mM Tris–HCl (pH-7.4) and 150 mM NaCl (SEC buffer). HiLoad 16/60 Superdex 200 pg column was equilibrated in the same SEC buffer. Proteins (2–5 mg/ml) were injected into the column and a flow rate of 1 ml/min was maintained throughout the chromatography. Fractions corresponding to the peaks in SEC were collected and observed in SDS PAGE. For calibrations of the SEC column, following gel filtration markers were used (molecular weight and corresponding elution volumes are indicated): Ferritin (440 kDa ~ 51 ml), Aldolase (158 kDa ~ 62 ml), Ovalbumin (43 kDa ~ 76 ml), Carbonic anhydrase (29 kDa ~ 84 ml), Ribonuclease A (13.7 kDa ~ 92 ml).

### Near UV CD spectroscopy

Near UV CD spectra were recorded by Jasco J-815 spectrophotometer. Spectra were recorded for 20 μM of proteins from 300 to 250 nm with a scan speed of 10 nm/min. 1 cm path length cuvette was used. Proteins were dialyzed and diluted in 10 mM sodium phosphate (pH-7.4) and 50 mM NaCl buffer. Buffer spectrum was subtracted from the protein CD spectra.

### ANS fluorescence spectroscopy

The fluorescence measurements were recorded using Jasco FP-6500 fluorimeter. ANS emission spectra were monitored from 400 to 600 nm with a scan speed of 50 nm/min, using a 1 cm path length cuvette. Both the protein and the ANS were prepared in 10 mM sodium phosphate (pH-7.4) and 50 mM NaCl buffer. 2 μM of protein was used and up to 30 μM ANS was incubated with the protein. The buffer spectrum was subtracted from the ANS and protein fluorescence spectra.

### Dynamic light scattering

DLS profile was obtained using a Malvern-Zetasizer nano ZS DLS instrument. The Proteins were concentrated to 2–5 mg/ml and filtered using a 0.22 micron filter to remove any aggregate. Molecular weight corresponding to the experimental hydrodynamic diameter was calculated using the software for the instrument.

### Homology modelling, ConSurf and WebLogo analysis, and molecular docking of ∆PcrG_(13–72)_ and PcrV

The spatial model of PcrV was generated by I-Tasser server using the structure of LcrV from *Yersinia pestis* (PDBID: 1R6F) as the template. The sequence of PcrV was loaded in the FASTA format to I-Tasser as the input file. Additional restrained, or templates were not assigned by the user. ∆PcrG_(13–72)_ is also modelled by I-Tasser using a threading approach, where the threading programmes of I-tasser assigned the top 10 threading templates. The best threading templates for ∆PcrG_(13–72)_ have PDBID: 2ZB9, 2Q24, 3SHG, 1GJS, 2B8I, 1LLW, 1ZBP, 2B8I, and 2R78 [22,23]. From the output file the best model was represented in PyMOL Molecular Graphics System [38]. The PDB file of the best model of PcrV generated by I-tasser was submitted as input to the ConSurf server. The default parameters of the server were used for the prediction. To generate the final model the output file- ConSurf modified PDB was loaded in PyMOL, and conservation code information in the script consurf_new.py was ran [26,38]. WebLogo server was used to generate sequence Logos of helix-7 and helix-12 of V-antigens and the sequences of corresponding helices of PcrV were aligned with these sequence Logos. MSA of helix-7 and helix-12 of proteins belonging to LcrV family was loaded as input [28]. To generate a model of ∆PcrG_(13–72)_-PcrV interaction by molecular docking, The PDB file of PcrV was loaded as the receptor and ∆PcrG_(13–72)_ was loaded as the ligand to the Z-Dock server (version ZD 3.0.2) [36]. Finally, best model was selected from top 5 predictions and represented using Jmol and PyMOL [38,39]. All the models were further checked and validated by PROCHECK [24].

### Multiple sequence alignment, disorder and coiled-coil prediction

Multiple sequence alignment profile of PcrG and PcrV with their respective homologs, were generated using MultAlin interface [27]. Disordered region in PcrG were predicted by PrDOS, DisEMBL 1.5 and Disopred version 2.0 [31-33]. COILS/PCOILS server from expasy predicted the intramolecular coiled-coil region within PcrG [34].

### Proteolytic digestion of PcrV, PcrG-PcrV with α-chymotrypsin and PcrG with elastase

PcrV, PcrG-PcrV and PcrG were dialyzed in 10 mM hepes (pH-7.4) and 150 mM NaCl. Proteases α-chymotrypsin and elastase from Proti-Ace Kit of Hampton research was diluted to 0.02 μg/μl by Proti-Ace dilution buffer from an initial stock of 1 μg/μl of protease in deionised water. 1 μl of protease from the diluted stock (0.02 μg/μl) was used for 10 μg of protein. Proteolytic digestion was carried out at 37°C for different time points and SDS PAGE sample lysis buffer was used to stop the protease activity. Finally the products obtained after digestion, were analyzed by SDS PAGE.

### Native mass spectrometry

Native proteins as well as the proteolytic digestion products of PcrV, PcrG-PcrV, and PcrG were diluted to 0.5-1.0 mg/ml concentration using HPLC water and immediately spotted on the MALDI target plate. Proteolytic digestions were stopped by trifluoroacetic acid present in the α-cyano hydroxyl cinnamic acid (CHCA) matrix. Mass was analyzed using an Applied Biosystem 4700 Proteomics Analyser 170.

### MS/MS sequence analysis of different fragments of PcrG, PcrV and PcrG-PcrV

After a specific time point, the digestion fragments of PcrG, PcrV and PcrG-PcrV were separated by SDS PAGE. The gel was stained with coomassie R-250 and washed with distilled water. The bands were excised from SDS PAGE and In-Gel tryptic digestion was carried out using trypsin gold from Promega. The “In-Gel tryptic digestion and MS/MS analysis” protocol provided by Promega was followed. Only exceptions to protocol are: the proteins were eluted in 40 mM ammonium bicarbonate, 10% acetonitrile and 0.3% trifluoroacetic acid buffer. During MS/MS analysis CHCA was used as matrix and prominent peaks were identified by MS after trypsinolysis. Peptides corresponding to the prominent peaks were further fragmented by laser and precise peptide fragments were sequenced using MSDB database of MASCOT search engine, using GPS Explorer Software (version 3.6) [40].

### Chemical crosslinking

Chemical crosslinking was performed using water soluble crosslinker EGS-sulfonate. The proteins were dialyzed in 10 mM hepes (pH-7.4) and 150 mM NaCl. The cross linking reactions were carried out at room temperature for 30 minutes with 0.5 mM, 1 mM, and 2 mM of EGS-sulfonate, and finally stopped by addition of sample lysis buffer of SDS PAGE. The crosslinked proteins were analyzed in 12% SDS PAGE.

### Surface plasmon resonance

Surface plasmon resonance binding analysis was performed by using a BIACORE 3000 systems and NTA sensor chip (GE Healthcare Life Sciences). Initially, both the flow cells (experiment and the reference cell) were thoroughly washed, and equilibrated with running buffer. Nickel chloride solution was charged in the experiment cell for the binding of nickel to the NTA sensor chip. According to the instruction manual of GE Healthcare Life Sciences for Biacore systems, the reference cell was maintained as non activated flow cell and treated with similar concentration of analyte as used in the experiment cell. Histidine tag PcrG and ∆PcrG_(13–72)_ were used as ligands and immobilized on the Ni-NTA surface in experiment cell. PcrV and its deletion mutants were used as analytes. All the proteins were dialyzed in the running buffer (10 mM hepes (pH-7.4), 150 mM NaCl and 50 μM EDTA). Very low concentration of EDTA was recommended in the running buffer to minimize non specific binding. In absence of the ligand, analyte showed negligible interaction with the activated flow cell. 20 μl of 50–100 μg/ml concentration of the ligand was charged for initial coupling to Ni-NTA sensor chip and unbound ligand was thoroughly washed by the running buffer. Binding kinetics were determined by passing PcrV and ∆PcrV (s) (deletion mutants) at different concentration ranging from 10 nM to 400 nM over PcrG and ∆PcrG_(13–72)_. A flow rate of 5 μl/min and temperature of 25°C was maintained throughout the experiment. Binding constants and forward and backward rates of reactions were determined by BIAevaluation software version 4.1, using a Langmuir binding model. In all the cases, the reference cell was subtracted from the experiment cell by the software to eliminate any RU change occurring due to non specific binding.

### Availability of supporting data

The data sets supporting the results of this article are included within the article (and its additional files). The mass spectrometry profiles and MS/MS sequence analysis of proteolytically digested protein fragments are given in Additional files 4, 5, 6, 7, 10, and 11. The PDB files and corresponding PROCHECK analysis of the models of the proteins are given in Additional files 1, 2, 14, 15, 16 and 17.

## Abbreviations

TTSS: Type three secretion system; ANS- 8: Anilinonapthalene-1-sulfonate; DLS: Dynamic light scattering; MSA: Multiple sequence alignment; SPR: Surface plasmon resonance; SEC: Size exclusion chromatography; EGS: Ethylene glycol bis [succinimidyl succinate]; Ni-NTA: Nickel-nitrilotriacetic acid; CPD: Cysteine protease domain; CHCA: α-cyano hydroxyl cinnamic acid.

## Competing interests

The authors declare that they have no competing interests.

## Authors’ contributions

SDatta conceived the entire idea of the work, designed, suggested the experiments, analyzed the experimental results, and drafted the manuscript. AB framed the idea of the work, designed and carried out all the experiments, and prepared the manuscript. UD and SDey designed and purified the proteins and participated in the downstream experiments with AB. All the authors have read and approved the final manuscript.

## Supplementary Material

Additional file 1 **Homology model of PcrV.** PDB file of the homology model of PcrV was provided according to the editorial requirement. Click here for file

Additional file 2 **Ramachandran Plot for homology model of PcrV.** For Validation of homology model of PcrV, PROCHECK server was used, which generated the corresponding Ramachandran Plot showing residues in the most favoured, allowed and disallowed region in the model. Click here for file

Additional file 3 **Multiple sequence alignment of PcrV.** Identity and similarity in the sequence of PcrV and its homologs (hydrophilic translocators of Ysc family) are shown. Click here for file

Additional file 4 **MS/MS sequence profile of 1st proteolytic digestion fragment of PcrV.** Sequence of the approximate region corresponding to the 1st proteolytic digestion fragment of PcrV, as revealed by MS/MS sequence analysis. Click here for file

Additional file 5 **MS/MS sequence profile of 2nd proteolytic digestion fragment of PcrV.** Almost entire sequence of the region corresponding to the 2nd proteolytic digestion fragment of PcrV, as revealed by MS/MS sequence analysis. Click here for file

Additional file 6 **Mass spectrometry profile of specifically protected fragment of PcrV (in presence of PcrG) during proteolytic digestion.** Molecular weight of the protected fragment of PcrV in presence of PcrG was estimated by mass spectrometry. Click here for file

Additional file 7 **MS/MS sequence profile of specifically protected fragment of PcrV, in presence of PcrG during proteolytic digestion.** Almost entire sequence of the region corresponding to the specifically protected fragment of PcrV in presence of PcrG during proteolytic digestion, as revealed by MS/MS sequence analysis. Click here for file

Additional file 8 **Native PAGE showing oligomeric state of ∆PcrV**_**(128–294)**_**, and heterodimeric state of PcrG-∆PcrV**_**(128–294).**_ Both ∆PcrV_(128–294)_ and PcrG-∆PcrV_(128–294)_ complex were run on the native PAGE. Since, there is no denaturation of the proteins the greater migration of PcrG-∆PcrV_(128–294)_ compared to ∆PcrV_(128–294)_, shows reversion of the oligomeric state to a lower order species, may be to a heterodimeric form. Click here for file

Additional file 9 **Disordered region of PcrG predicted by DisEMBL 1.5, Disopred version 2.0.** DisEMBL 1.5, Disopred version 2.0 disorder prediction servers predicting the disordered regions (regions lacking proper secondary structure) within PcrG, by various algorithms used by these servers. Click here for file

Additional file 10 **Mass spectrometry profile of the proteolytically digested fragment of PcrG.** Molecular weight of the proteolytically digested fragment of PcrG was estimated by mass spectrometry. Click here for file

Additional file 11 **MS/MS sequence profile of proteolytically digested fragment of PcrG.** Sequence of the approximate region corresponding to the digested fragment of PcrG, as revealed by MS/MS sequence analysis. Click here for file

Additional file 12 **Multiple sequence alignment of PcrG.** Identity and similarity in the sequence of PcrG and its homologs, are shown. Click here for file

Additional file 13 **Coiled-coil regions of PcrG predicted by COILS/PCOILS.** Probablity of occurrence of intramolecular coiled-coil regions (essential for protein-protein interaction) within PcrG, predicted by COILS/PCOILS server, is shown. Click here for file

Additional file 14 **Model of ∆PcrG**_**(13–72)**_**.** PDB file of the model of ∆PcrG_(13–72)_ was provided according to the editorial requirement. Click here for file

Additional file 15 **Ramachandran Plot for the model of ∆PcrG**_**(13–72)**_**.** For Validation of the model of ∆PcrG_(13–72)_, PROCHECK server was used, which generated the corresponding Ramachandran Plot showing residues in the most favoured, allowed and disallowed region in the model. Click here for file

Additional file 16 **Model of ∆PcrG**_**(13–72)**_**-PcrV.** PDB file of the Model of ∆PcrG_(13–72)_-PcrV was provided according to the editorial requirement. Click here for file

Additional file 17 **Ramachandran Plot for the model of ∆PcrG**_**(13–72)**_**-PcrV generated by molecular docking.** For Validation of the model of ∆PcrG_(13–72)_-PcrV, PROCHECK server was used, which generated the corresponding Ramachandran Plot showing residues in the most favoured, allowed and disallowed region in the model. Click here for file
