# Supplementary figures and images for: PcrG protects the two long helical oligomerization domains of PcrV, by an interaction mediated by the intramolecular coiled-coil region of PcrG
Source: BMC Struct Biol. 2014 Jan 24;14:5. doi: 10.1186/1472-6807-14-5 (PMC3904411; doi:10.1186/1472-6807-14-5)

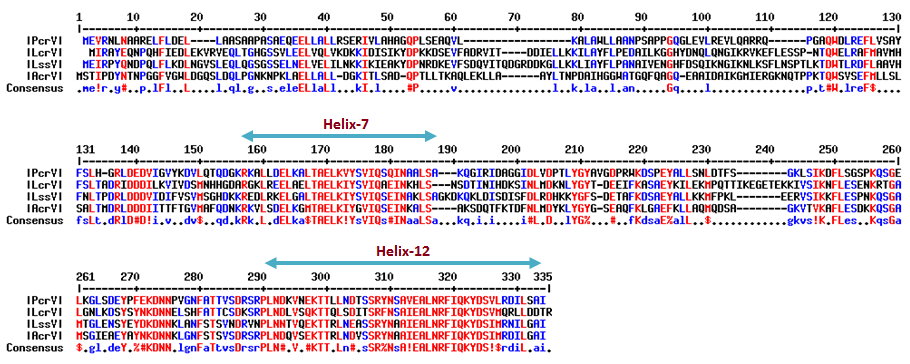

Supplement: Additional file 3 — Multiple sequence alignment of PcrV. Identity and similarity in the sequence of PcrV and its homologs (hydrophilic translocators of Ysc family) are shown. [file 1472-6807-14-5-S3.tiff]

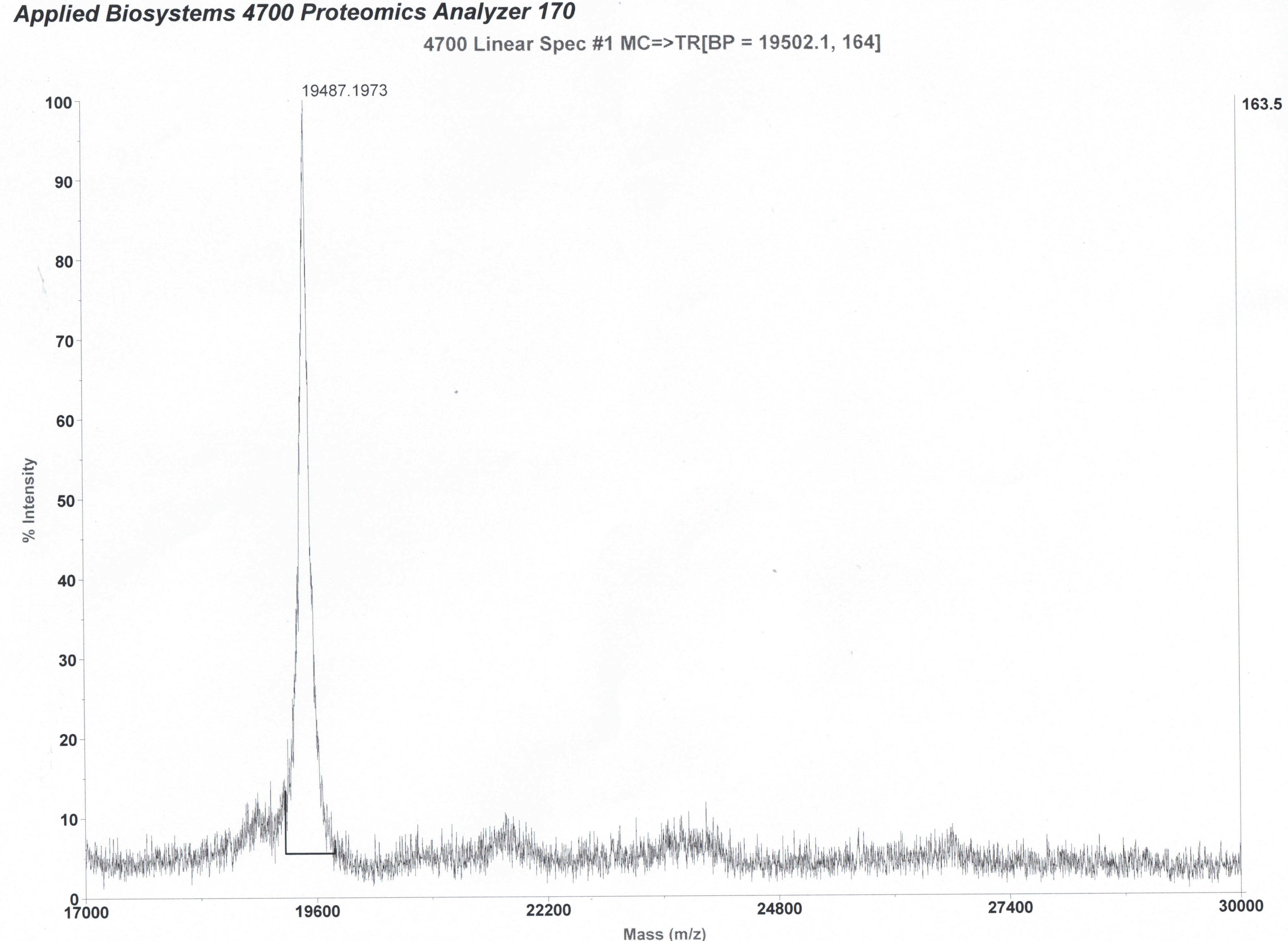

Supplement: Additional file 6 — Mass spectrometry profile of specifically protected fragment of PcrV (in presence of PcrG) during proteolytic digestion. Molecular weight of the protected fragment of PcrV in presence of PcrG was estimated by mass spectrometry. [file 1472-6807-14-5-S6.tiff]

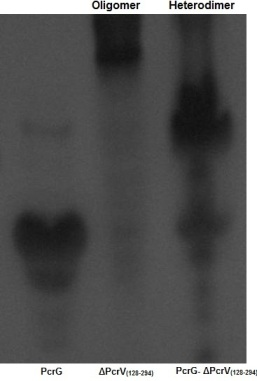

Supplement: Additional file 8 — Native PAGE showing oligomeric state of ∆PcrV (128–294) , and heterodimeric state of PcrG-∆PcrV (128–294). Both ∆PcrV(128–294) and PcrG-∆PcrV(128–294) complex were run on the native PAGE. Since, there is no denaturation of the proteins the greater migration of PcrG-∆PcrV(128–294) compared to ∆PcrV(128–294), shows reversion of the oligomeric state to a lower order species, may be to a heterodimeric form. [file 1472-6807-14-5-S8.tiff]

**DisEMBL 1.5**

**
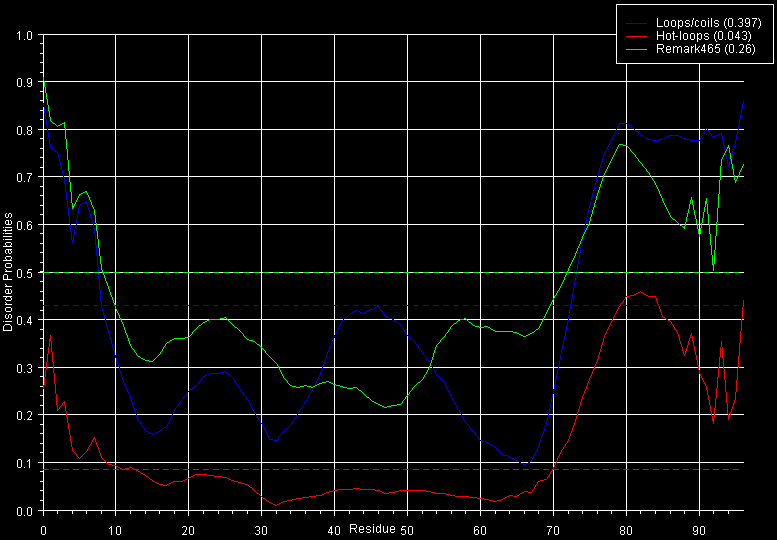
**

**
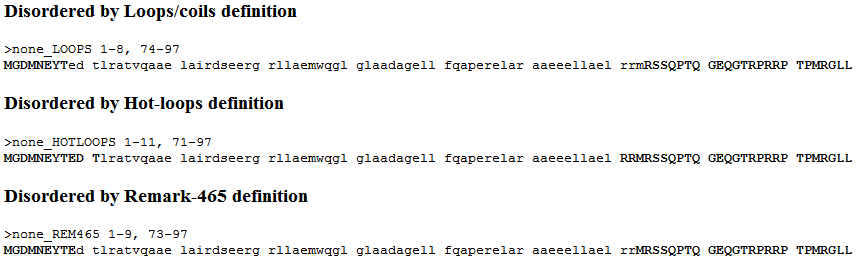
**

**Disopred**

**
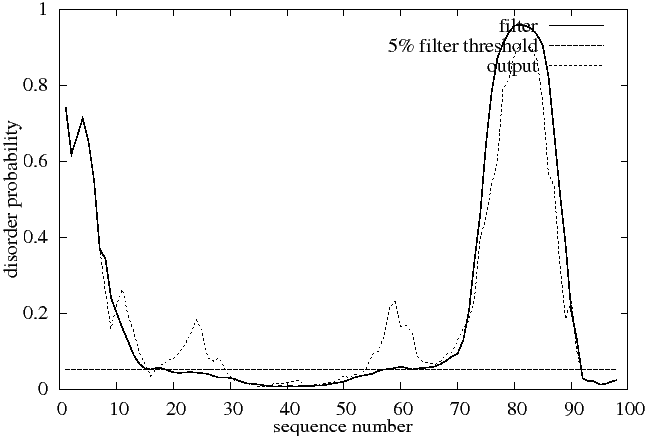
**

**
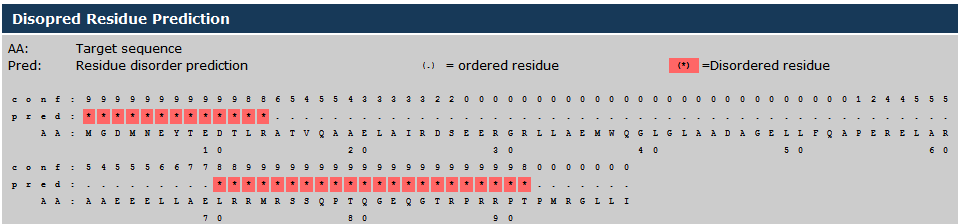
**

Supplement: Additional file 9 — Disordered region of PcrG predicted by DisEMBL 1.5, Disopred version 2.0. DisEMBL 1.5, Disopred version 2.0 disorder prediction servers predicting the disordered regions (regions lacking proper secondary structure) within PcrG, by various algorithms used by these servers. [file 1472-6807-14-5-S9.docx]

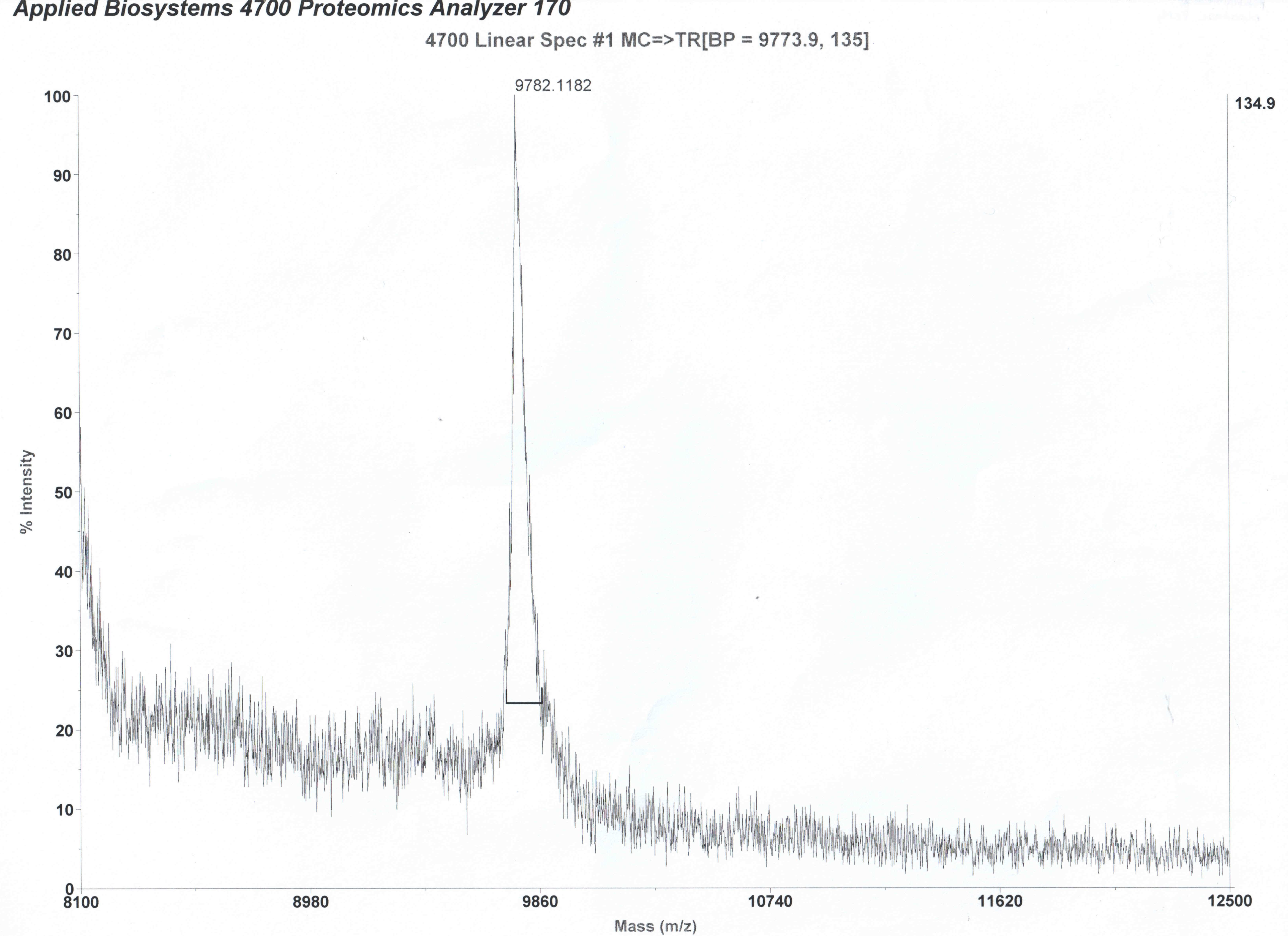

Supplement: Additional file 10 — Mass spectrometry profile of the proteolytically digested fragment of PcrG. Molecular weight of the proteolytically digested fragment of PcrG was estimated by mass spectrometry. [file 1472-6807-14-5-S10.tiff]

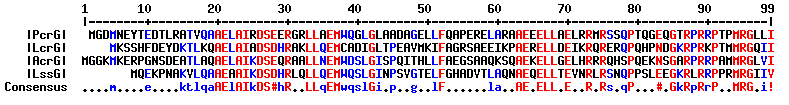

Supplement: Additional file 12 — Multiple sequence alignment of PcrG. Identity and similarity in the sequence of PcrG and its homologs, are shown. [file 1472-6807-14-5-S12.tiff]

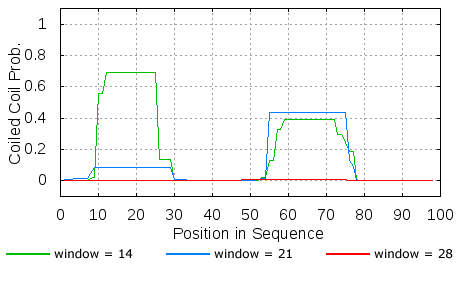

Supplement: Additional file 13 — Coiled-coil regions of PcrG predicted by COILS/PCOILS. Probablity of occurrence of intramolecular coiled-coil regions (essential for protein-protein interaction) within PcrG, predicted by COILS/PCOILS server, is shown. [file 1472-6807-14-5-S13.tiff]
