# Supplementary material for: PcrG protects the two long helical oligomerization domains of PcrV, by an interaction mediated by the intramolecular coiled-coil region of PcrG
Source: BMC Struct Biol. 2014 Jan 24;14:5. doi: 10.1186/1472-6807-14-5 (PMC3904411; doi:10.1186/1472-6807-14-5)
Supplement: Additional file 5 — MS/MS sequence profile of 2nd proteolytic digestion fragment of PcrV. Almost entire sequence of the region corresponding to the 2nd proteolytic digestion fragment of PcrV, as revealed by MS/MS sequence analysis. [file 1472-6807-14-5-S5.docx]

# **Mascot Search Results**

### **Protein View**

Match to: **G83432** Score: **242** Expect: **4.8e-019
type III secretion protein PcrV PA1706 [imported] - Pseudomonas aeruginosa (strain PAO1)**Nominal mass (M_r_): **32264**; Calculated pI value: **5.03**NCBI BLAST search of [G83432](http://www.ncbi.nlm.nih.gov/blast/Blast.cgi?ALIGNMENTS=50&amp;ALIGNMENT_VIEW=Pairwise&amp;AUTO_FORMAT=Semiauto&amp;CDD_SEARCH=on&amp;CLIENT=web&amp;COMPOSITION_BASED_STATISTICS=on&amp;DATABASE=nr&amp;DESCRIPTIONS=100&amp;ENTREZ_QUERY=(none)&amp;EXPECT=10&amp;FILTER=L&amp;FORMAT_BLOCK_ON_RESPAGE=None&amp;FORMAT_OBJECT=Alignment&amp;FORMAT_TYPE=HTML&amp;GAPCOSTS=11+1&amp;I_THRESH=0.001&amp;LAYOUT=TwoWindows&amp;MATRIX_NAME=BLOSUM62&amp;NCBI_GI=on&amp;PAGE=Proteins&amp;PROGRAM=blastp&amp;QUERY=MEVRNLNAARELFLDELLAASAAPASAEQEELLALLRSERIVLAHAGQPLSEAQVLKALAWLLAANPSAPPGQGLEVLREVLQARRQPGAQWDLREFLVSAYFSLHGRLDEDVIGVYKDVLQTQDGKRKALLDELKALTAELKVYSVIQSQINAALSAKQGIRIDAGGIDLVDPTLYGYAVGDPRWKDSPEYALLSNLDTFSGKLSIKDFLSGSPKQSGELKGLSDEYPFEKDNNPVGNFATTVSDRSRPLNDKVNEKTTLLNDTSSRYNSAVEALNRFIQKYDSVLRDILSAI&amp;SERVICE=plain&amp;SET_DEFAULTS.x=9&amp;SET_DEFAULTS.y=5&amp;SHOW_OVERVIEW=on&amp;WORD_SIZE=3&amp;END_OF_HTTPGET=Yes) against nr
Unformatted [sequence string](http://iicbgps/mascot/cgi/getseq.pl?MSDB+G83432+seq) for pasting into other applications

Taxonomy: [Pseudomonas aeruginosa](http://www.ncbi.nlm.nih.gov/htbin-post/Taxonomy/wgetorg?lvl=0&amp;lin=f&amp;id=287)
Links to retrieve other entries containing this sequence from NCBI Entrez:
[O30527_PSEAE](http://www.ncbi.nlm.nih.gov/entrez/eutils/efetch.fcgi?db=protein&amp;retmode=html&amp;rettype=gp&amp;id=O30527_PSEAE) from [Pseudomonas aeruginosa](http://www.ncbi.nlm.nih.gov/htbin-post/Taxonomy/wgetorg?lvl=0&amp;lin=f&amp;id=287)
[AAG05095](http://www.ncbi.nlm.nih.gov/entrez/eutils/efetch.fcgi?db=protein&amp;retmode=html&amp;rettype=gp&amp;id=AAG05095) from [Pseudomonas aeruginosa PAO1](http://www.ncbi.nlm.nih.gov/htbin-post/Taxonomy/wgetorg?lvl=0&amp;lin=f&amp;id=208964)
[AAC45935](http://www.ncbi.nlm.nih.gov/entrez/eutils/efetch.fcgi?db=protein&amp;retmode=html&amp;rettype=gp&amp;id=AAC45935) from [Pseudomonas aeruginosa](http://www.ncbi.nlm.nih.gov/htbin-post/Taxonomy/wgetorg?lvl=0&amp;lin=f&amp;id=287)
[AAO91771](http://www.ncbi.nlm.nih.gov/entrez/eutils/efetch.fcgi?db=protein&amp;retmode=html&amp;rettype=gp&amp;id=AAO91771) from [Pseudomonas aeruginosa](http://www.ncbi.nlm.nih.gov/htbin-post/Taxonomy/wgetorg?lvl=0&amp;lin=f&amp;id=287)

Fixed modifications: Carbamidomethyl (C)
Variable modifications: Oxidation (M)
Cleavage by Trypsin: cuts C-term side of KR unless next residue is P
Sequence Coverage: **28%**Matched peptides shown in **Bold Red

 1** MEVRNLNAAR ELFLDELLAA SAAPASAEQE ELLALLRSER **IVLAHAGQPL
 51 SEAQVLKALA WLLAANPSAP PGQGLEVLR**E VLQAR**RQPGA QWDLREFLVS
 101 AYFSLHGR**LD EDVIGVYKDV LQTQDGKRKA LLDELK**ALTA ELKVYSVIQS
 151 QINAALSAK**Q GIRIDAGGID LVDPTLYGYA VGDPRWKDSP EYALLSNLDT
 **201** FSGKLSIKDF LSGSPKQSGE LKGLSDEYPF EKDNNPVGNF ATTVSDRSRP
 **251** LNDKVNEKTT LLNDTSSRYN SAVEALNRFI QKYDSVLRDI LSAI

 
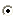
Residue Number 
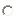
Increasing Mass 
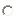
Decreasing Mass

**Start - End Observed Mr(expt) Mr(calc) Delta Miss Sequence
 41 - 57 1774.0123 1773.0050 1773.0148 -0.0098 0 R.IVLAHAGQPLSEAQVLK.A** ([No match](http://iicbgps/mascot/cgi/peptide_view.pl?file=../data/20120828/F006573.dat&amp;query=38&amp;hit=1))
 **41 - 79 3999.2942 3998.2869 3998.2468 0.0401 1 R.IVLAHAGQPLSEAQVLKALAWLLAANPSAPPGQGLEVLR.E** ([No match](http://iicbgps/mascot/cgi/peptide_view.pl?file=../data/20120828/F006573.dat&amp;query=74&amp;hit=1))
 **41 - 79 3999.2942 3998.2869 3998.2468 0.0401 1 R.IVLAHAGQPLSEAQVLKALAWLLAANPSAPPGQGLEVLR.E** ([No match](http://iicbgps/mascot/cgi/peptide_view.pl?file=../data/20120828/F006573.dat&amp;query=75&amp;hit=1))
 **58 - 79 2244.2349 2243.2276 2243.2426 -0.0149 0 K.ALAWLLAANPSAPPGQGLEVLR.E** ([Ions score 70](http://iicbgps/mascot/cgi/peptide_view.pl?file=../data/20120828/F006573.dat&amp;query=49&amp;hit=1))
 **58 - 79 2244.2349 2243.2276 2243.2426 -0.0149 0 K.ALAWLLAANPSAPPGQGLEVLR.E** ([No match](http://iicbgps/mascot/cgi/peptide_view.pl?file=../data/20120828/F006573.dat&amp;query=50&amp;hit=1))
 **86 - 95 1226.6394 1225.6321 1225.6316 0.0005 1 R.RQPGAQWDLR.E** ([Ions score 14](http://iicbgps/mascot/cgi/peptide_view.pl?file=../data/20120828/F006573.dat&amp;query=17&amp;hit=1))
 **86 - 95 1226.6394 1225.6321 1225.6316 0.0005 1 R.RQPGAQWDLR.E** ([No match](http://iicbgps/mascot/cgi/peptide_view.pl?file=../data/20120828/F006573.dat&amp;query=18&amp;hit=1))
 **87 - 95 1070.5505 1069.5432 1069.5305 0.0128 0 R.QPGAQWDLR.E** ([No match](http://iicbgps/mascot/cgi/peptide_view.pl?file=../data/20120828/F006573.dat&amp;query=9&amp;hit=1))
 **87 - 108 2577.2896 2576.2823 2576.2924 -0.0100 1 R.QPGAQWDLREFLVSAYFSLHGR.L** ([Ions score 38](http://iicbgps/mascot/cgi/peptide_view.pl?file=../data/20120828/F006573.dat&amp;query=56&amp;hit=1))
 **87 - 108 2577.2896 2576.2823 2576.2924 -0.0100 1 R.QPGAQWDLREFLVSAYFSLHGR.L** ([No match](http://iicbgps/mascot/cgi/peptide_view.pl?file=../data/20120828/F006573.dat&amp;query=57&amp;hit=1))
 **96 - 108 1525.7732 1524.7659 1524.7724 -0.0065 0 R.EFLVSAYFSLHGR.L** ([Ions score 48](http://iicbgps/mascot/cgi/peptide_view.pl?file=../data/20120828/F006573.dat&amp;query=31&amp;hit=1))
 **96 - 108 1525.7732 1524.7659 1524.7724 -0.0065 0 R.EFLVSAYFSLHGR.L** ([No match](http://iicbgps/mascot/cgi/peptide_view.pl?file=../data/20120828/F006573.dat&amp;query=32&amp;hit=1))
 **137 - 159 2418.3450 2417.3377 2417.3529 -0.0152 1 K.ALTAELKVYSVIQSQINAALSAK.Q** ([No match](http://iicbgps/mascot/cgi/peptide_view.pl?file=../data/20120828/F006573.dat&amp;query=51&amp;hit=1))
 **144 - 159 1691.9209 1690.9136 1690.9253 -0.0117 0 K.VYSVIQSQINAALSAK.Q** ([No match](http://iicbgps/mascot/cgi/peptide_view.pl?file=../data/20120828/F006573.dat&amp;query=35&amp;hit=1))



>P1;G83432
type III secretion protein PcrV PA1706 [imported] - Pseudomonas aeruginosa (strain PAO1)
C;Species G83432: Pseudomonas aeruginosa
C;Species O30527_PSEAE: Pseudomonas aeruginosa.
C;Species AAG05095: Pseudomonas aeruginosa PAO1
C;Species AAC45935: Pseudomonas aeruginosa
C;Species AAO91771: Pseudomonas aeruginosa
C;Date: 15-Sep-2000 #sequence_revision 15-Sep-2000 #text_change 09-Jul-2004
C;Accession: G83432
R;Stover, C.K.; Pham, X.Q.; Erwin, A.L.; Mizoguchi, S.D.; Warrener, P.; Hickey, M.J.; Brinkman, F.S.L.; Hufnagle, W.O.; Kowalik, D.J.; Lagrou, M.; Garber, R.L.; Goltry, L.; Tolentino, E.; Westbrook-Wadman, S.; Yuan, Y.; Brody, L.L.; Coulter, S.N.; Folger, K.R.; Kas, A.; Larbig, K.; Lim, R.M.; Smith, K.A.; Spencer, D.H.; Wong, G.K.S.; Wu, Z.; Paulsen, I.T.; Reizer, J.; Saier, M.H.; Hancock, R.E.W.; Lory, S.; Olson, M.V.
Nature 406, 959-964, 2000
A;Title: Complete genome sequence of Pseudomonas aeruginosa PA01, an opportunistic pathogen.
A;Reference number: A82950; MUID:20437337; PMID:10984043
A;Accession: G83432
A;Status: preliminary
A;Molecule type: DNA
A;Residues: 1-294
A;Cross-references: UNIPROT:O30527; UNIPARC:UPI00000D424F; GB:AE004597; GB:AE004091; NID:g9947671; PIDN:AAG05095.1; GSPDB:GN00131; PASP:PA1706
A;Experimental source: strain PAO1
C;Genetics:
A;Gene: pcrV; PA1706
C;SRCDB PIR2
C;IDN_TREMBL O30527_PSEAE;
C;IDN_GENBANK AAG05095; AAC45935; AAO91771;

| **Mascot:**  <http://www.matrixscience.com/> |
| --- |
