# Supplementary material for: PcrG protects the two long helical oligomerization domains of PcrV, by an interaction mediated by the intramolecular coiled-coil region of PcrG
Source: BMC Struct Biol. 2014 Jan 24;14:5. doi: 10.1186/1472-6807-14-5 (PMC3904411; doi:10.1186/1472-6807-14-5)
Supplement: Additional file 7 — MS/MS sequence profile of specifically protected fragment of PcrV, in presence of PcrG during proteolytic digestion. Almost entire sequence of the region corresponding to the specifically protected fragment of PcrV in presence of PcrG during proteolytic digestion, as revealed by MS/MS sequence analysis. [file 1472-6807-14-5-S7.docx]

# **Mascot Search Results**

### **Protein View**

Match to: **G83432** Score: **114** Expect: **3e-006
type III secretion protein PcrV PA1706 [imported] - Pseudomonas aeruginosa (strain PAO1)**Nominal mass (M_r_): **32264**; Calculated pI value: **5.03**NCBI BLAST search of [G83432](http://www.ncbi.nlm.nih.gov/blast/Blast.cgi?ALIGNMENTS=50&amp;ALIGNMENT_VIEW=Pairwise&amp;AUTO_FORMAT=Semiauto&amp;CDD_SEARCH=on&amp;CLIENT=web&amp;COMPOSITION_BASED_STATISTICS=on&amp;DATABASE=nr&amp;DESCRIPTIONS=100&amp;ENTREZ_QUERY=(none)&amp;EXPECT=10&amp;FILTER=L&amp;FORMAT_BLOCK_ON_RESPAGE=None&amp;FORMAT_OBJECT=Alignment&amp;FORMAT_TYPE=HTML&amp;GAPCOSTS=11+1&amp;I_THRESH=0.001&amp;LAYOUT=TwoWindows&amp;MATRIX_NAME=BLOSUM62&amp;NCBI_GI=on&amp;PAGE=Proteins&amp;PROGRAM=blastp&amp;QUERY=MEVRNLNAARELFLDELLAASAAPASAEQEELLALLRSERIVLAHAGQPLSEAQVLKALAWLLAANPSAPPGQGLEVLREVLQARRQPGAQWDLREFLVSAYFSLHGRLDEDVIGVYKDVLQTQDGKRKALLDELKALTAELKVYSVIQSQINAALSAKQGIRIDAGGIDLVDPTLYGYAVGDPRWKDSPEYALLSNLDTFSGKLSIKDFLSGSPKQSGELKGLSDEYPFEKDNNPVGNFATTVSDRSRPLNDKVNEKTTLLNDTSSRYNSAVEALNRFIQKYDSVLRDILSAI&amp;SERVICE=plain&amp;SET_DEFAULTS.x=9&amp;SET_DEFAULTS.y=5&amp;SHOW_OVERVIEW=on&amp;WORD_SIZE=3&amp;END_OF_HTTPGET=Yes) against nr
Unformatted [sequence string](http://iicbgps/mascot/cgi/getseq.pl?MSDB+G83432+seq) for pasting into other applications

Taxonomy: [Pseudomonas aeruginosa](http://www.ncbi.nlm.nih.gov/htbin-post/Taxonomy/wgetorg?lvl=0&amp;lin=f&amp;id=287)
Links to retrieve other entries containing this sequence from NCBI Entrez:
[O30527_PSEAE](http://www.ncbi.nlm.nih.gov/entrez/eutils/efetch.fcgi?db=protein&amp;retmode=html&amp;rettype=gp&amp;id=O30527_PSEAE) from [Pseudomonas aeruginosa](http://www.ncbi.nlm.nih.gov/htbin-post/Taxonomy/wgetorg?lvl=0&amp;lin=f&amp;id=287)
[AAG05095](http://www.ncbi.nlm.nih.gov/entrez/eutils/efetch.fcgi?db=protein&amp;retmode=html&amp;rettype=gp&amp;id=AAG05095) from [Pseudomonas aeruginosa PAO1](http://www.ncbi.nlm.nih.gov/htbin-post/Taxonomy/wgetorg?lvl=0&amp;lin=f&amp;id=208964)
[AAC45935](http://www.ncbi.nlm.nih.gov/entrez/eutils/efetch.fcgi?db=protein&amp;retmode=html&amp;rettype=gp&amp;id=AAC45935) from [Pseudomonas aeruginosa](http://www.ncbi.nlm.nih.gov/htbin-post/Taxonomy/wgetorg?lvl=0&amp;lin=f&amp;id=287)
[AAO91771](http://www.ncbi.nlm.nih.gov/entrez/eutils/efetch.fcgi?db=protein&amp;retmode=html&amp;rettype=gp&amp;id=AAO91771) from [Pseudomonas aeruginosa](http://www.ncbi.nlm.nih.gov/htbin-post/Taxonomy/wgetorg?lvl=0&amp;lin=f&amp;id=287)

Fixed modifications: Carbamidomethyl (C)
Variable modifications: Oxidation (M)
Cleavage by Trypsin: cuts C-term side of KR unless next residue is P
Sequence Coverage: **47%**Matched peptides shown in **Bold Red

 1** MEVRNLNAAR ELFLDELLAA SAAPASAEQE ELLALLRSER IVLAHAGQPL
 **51** SEAQVLKALA WLLAANPSAP PGQGLEVLRE VLQARRQPGA QWDLREFLVS
 **101** AYFSLHGRLD EDVIGVYKDV LQTQDGKRKA LLDELK**ALTA ELKVYSVIQS
 151 QINAALSAKQ GIRIDAGGID LVDPTLYGYA VGDPRWKDSP EYALLSNLDT
 201 FSGKLSIKDF LSGSPK**QSGE LK**GLSDEYPF EKDNNPVGNF ATTVSDR**SRP
 **251** LNDKVNEK**TT LLNDTSSRYN SAVEALNRFI QKYDSVLRDI LSAI**

 
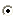
Residue Number 
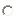
Increasing Mass 
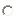
Decreasing Mass

**Start - End Observed Mr(expt) Mr(calc) Delta Miss Sequence
 137 - 159 2418.3140 2417.3067 2417.3529 -0.0462 1 K.ALTAELKVYSVIQSQINAALSAK.Q** ([No match](http://iicbgps/mascot/cgi/peptide_view.pl?file=../data/20121101/F008059.dat&amp;query=64&amp;hit=1))
 **144 - 159 1691.9095 1690.9022 1690.9253 -0.0231 0 K.VYSVIQSQINAALSAK.Q** ([No match](http://iicbgps/mascot/cgi/peptide_view.pl?file=../data/20121101/F008059.dat&amp;query=48&amp;hit=1))
 **160 - 185 2731.3403 2730.3330 2730.3976 -0.0646 1 K.QGIRIDAGGIDLVDPTLYGYAVGDPR.W** ([No match](http://iicbgps/mascot/cgi/peptide_view.pl?file=../data/20121101/F008059.dat&amp;query=65&amp;hit=1))
 **164 - 185 2277.1001 2276.0928 2276.1324 -0.0396 0 R.IDAGGIDLVDPTLYGYAVGDPR.W** ([No match](http://iicbgps/mascot/cgi/peptide_view.pl?file=../data/20121101/F008059.dat&amp;query=62&amp;hit=1))
 **186 - 204 2171.0295 2170.0222 2170.0582 -0.0360 1 R.WKDSPEYALLSNLDTFSGK.L** ([No match](http://iicbgps/mascot/cgi/peptide_view.pl?file=../data/20121101/F008059.dat&amp;query=59&amp;hit=1))
 **205 - 216 1291.7078 1290.7005 1290.7183 -0.0178 1 K.LSIKDFLSGSPK.Q** ([No match](http://iicbgps/mascot/cgi/peptide_view.pl?file=../data/20121101/F008059.dat&amp;query=25&amp;hit=1))
 **205 - 216 1291.7078 1290.7005 1290.7183 -0.0178 1 K.LSIKDFLSGSPK.Q** ([No match](http://iicbgps/mascot/cgi/peptide_view.pl?file=../data/20121101/F008059.dat&amp;query=26&amp;hit=1))
 **223 - 247 2772.2112 2771.2039 2771.2674 -0.0635 1 K.GLSDEYPFEKDNNPVGNFATTVSDR.S** ([No match](http://iicbgps/mascot/cgi/peptide_view.pl?file=../data/20121101/F008059.dat&amp;query=66&amp;hit=1))
 **233 - 247 1606.7246 1605.7173 1605.7383 -0.0209 0 K.DNNPVGNFATTVSDR.S** ([No match](http://iicbgps/mascot/cgi/peptide_view.pl?file=../data/20121101/F008059.dat&amp;query=44&amp;hit=1))
 **259 - 278 2225.0720 2224.0647 2224.1083 -0.0436 1 K.TTLLNDTSSRYNSAVEALNR.F** ([No match](http://iicbgps/mascot/cgi/peptide_view.pl?file=../data/20121101/F008059.dat&amp;query=61&amp;hit=1))
 **269 - 278 1136.5563 1135.5490 1135.5621 -0.0131 0 R.YNSAVEALNR.F** ([No match](http://iicbgps/mascot/cgi/peptide_view.pl?file=../data/20121101/F008059.dat&amp;query=17&amp;hit=1))
 **269 - 282 1652.8517 1651.8444 1651.8681 -0.0237 1 R.YNSAVEALNRFIQK.Y** ([No match](http://iicbgps/mascot/cgi/peptide_view.pl?file=../data/20121101/F008059.dat&amp;query=47&amp;hit=1))
 **279 - 288 1268.6801 1267.6728 1267.6924 -0.0196 1 R.FIQKYDSVLR.D** ([No match](http://iicbgps/mascot/cgi/peptide_view.pl?file=../data/20121101/F008059.dat&amp;query=23&amp;hit=1))
 **283 - 294 1364.7279 1363.7206 1363.7347 -0.0141 1 K.YDSVLRDILSAI.-** ([No match](http://iicbgps/mascot/cgi/peptide_view.pl?file=../data/20121101/F008059.dat&amp;query=33&amp;hit=1))



>P1;G83432
type III secretion protein PcrV PA1706 [imported] - Pseudomonas aeruginosa (strain PAO1)
C;Species G83432: Pseudomonas aeruginosa
C;Species O30527_PSEAE: Pseudomonas aeruginosa.
C;Species AAG05095: Pseudomonas aeruginosa PAO1
C;Species AAC45935: Pseudomonas aeruginosa
C;Species AAO91771: Pseudomonas aeruginosa
C;Date: 15-Sep-2000 #sequence_revision 15-Sep-2000 #text_change 09-Jul-2004
C;Accession: G83432
R;Stover, C.K.; Pham, X.Q.; Erwin, A.L.; Mizoguchi, S.D.; Warrener, P.; Hickey, M.J.; Brinkman, F.S.L.; Hufnagle, W.O.; Kowalik, D.J.; Lagrou, M.; Garber, R.L.; Goltry, L.; Tolentino, E.; Westbrook-Wadman, S.; Yuan, Y.; Brody, L.L.; Coulter, S.N.; Folger, K.R.; Kas, A.; Larbig, K.; Lim, R.M.; Smith, K.A.; Spencer, D.H.; Wong, G.K.S.; Wu, Z.; Paulsen, I.T.; Reizer, J.; Saier, M.H.; Hancock, R.E.W.; Lory, S.; Olson, M.V.
Nature 406, 959-964, 2000
A;Title: Complete genome sequence of Pseudomonas aeruginosa PA01, an opportunistic pathogen.
A;Reference number: A82950; MUID:20437337; PMID:10984043
A;Accession: G83432
A;Status: preliminary
A;Molecule type: DNA
A;Residues: 1-294
A;Cross-references: UNIPROT:O30527; UNIPARC:UPI00000D424F; GB:AE004597; GB:AE004091; NID:g9947671; PIDN:AAG05095.1; GSPDB:GN00131; PASP:PA1706
A;Experimental source: strain PAO1
C;Genetics:
A;Gene: pcrV; PA1706
C;SRCDB PIR2
C;IDN_TREMBL O30527_PSEAE;
C;IDN_GENBANK AAG05095; AAC45935; AAO91771;

| **Mascot:**  <http://www.matrixscience.com/> |
| --- |
