# Supplementary material for: PcrG protects the two long helical oligomerization domains of PcrV, by an interaction mediated by the intramolecular coiled-coil region of PcrG
Source: BMC Struct Biol. 2014 Jan 24;14:5. doi: 10.1186/1472-6807-14-5 (PMC3904411; doi:10.1186/1472-6807-14-5)
Supplement: Additional file 11 — MS/MS sequence profile of proteolytically digested fragment of PcrG. Sequence of the approximate region corresponding to the digested fragment of PcrG, as revealed by MS/MS sequence analysis. [file 1472-6807-14-5-S11.docx]

# **Mascot Search Results**

### **Protein View**

Match to: **F83432** Score: **81** Expect: **0.0058
regulator in type III secretion PA1705 [imported] - Pseudomonas aeruginosa (strain PAO1)**Nominal mass (M_r_): **10989**; Calculated pI value: **4.80**NCBI BLAST search of [F83432](http://www.ncbi.nlm.nih.gov/blast/Blast.cgi?ALIGNMENTS=50&amp;ALIGNMENT_VIEW=Pairwise&amp;AUTO_FORMAT=Semiauto&amp;CDD_SEARCH=on&amp;CLIENT=web&amp;COMPOSITION_BASED_STATISTICS=on&amp;DATABASE=nr&amp;DESCRIPTIONS=100&amp;ENTREZ_QUERY=(none)&amp;EXPECT=10&amp;FILTER=L&amp;FORMAT_BLOCK_ON_RESPAGE=None&amp;FORMAT_OBJECT=Alignment&amp;FORMAT_TYPE=HTML&amp;GAPCOSTS=11+1&amp;I_THRESH=0.001&amp;LAYOUT=TwoWindows&amp;MATRIX_NAME=BLOSUM62&amp;NCBI_GI=on&amp;PAGE=Proteins&amp;PROGRAM=blastp&amp;QUERY=MGDMNEYTEDTLRATVQAAELAIRDSEERGRLLAEMWQGLGLAADAGELLFQAPERELARAAEEELLAELRRMRSSQPTQGEQGTRPRRPTPMRGLLI&amp;SERVICE=plain&amp;SET_DEFAULTS.x=9&amp;SET_DEFAULTS.y=5&amp;SHOW_OVERVIEW=on&amp;WORD_SIZE=3&amp;END_OF_HTTPGET=Yes) against nr
Unformatted [sequence string](http://iicbgps/mascot/cgi/getseq.pl?MSDB+F83432+seq) for pasting into other applications

Taxonomy: [Pseudomonas aeruginosa](http://www.ncbi.nlm.nih.gov/htbin-post/Taxonomy/wgetorg?lvl=0&amp;lin=f&amp;id=287)
Links to retrieve other entries containing this sequence from NCBI Entrez:
[Q9I326_PSEAE](http://www.ncbi.nlm.nih.gov/entrez/eutils/efetch.fcgi?db=protein&amp;retmode=html&amp;rettype=gp&amp;id=Q9I326_PSEAE) from [Pseudomonas aeruginosa](http://www.ncbi.nlm.nih.gov/htbin-post/Taxonomy/wgetorg?lvl=0&amp;lin=f&amp;id=287)
[AAG05094](http://www.ncbi.nlm.nih.gov/entrez/eutils/efetch.fcgi?db=protein&amp;retmode=html&amp;rettype=gp&amp;id=AAG05094) from [Pseudomonas aeruginosa PAO1](http://www.ncbi.nlm.nih.gov/htbin-post/Taxonomy/wgetorg?lvl=0&amp;lin=f&amp;id=208964)

Fixed modifications: Carbamidomethyl (C)
Variable modifications: Oxidation (M)
Cleavage by Trypsin: cuts C-term side of KR unless next residue is P
Sequence Coverage: **62%**Matched peptides shown in **Bold Red

 1** MGDMNEYTED TLRATVQAAE LAIRDSEERG R**LLAEMWQGL GLAADAGELL
 51 FQAPERELAR AAEEELLAEL RR**MR**SSQPTQ GEQGTRPRRP TPMR**GLLI

 
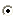
Residue Number 
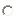
Increasing Mass 
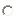
Decreasing Mass

**Start - End Observed Mr(expt) Mr(calc) Delta Miss Sequence
 32 - 56 2699.2026 2698.1953 2698.3788 -0.1834 0 R.LLAEMWQGLGLAADAGELLFQAPER.E** ([No match](http://iicbgps/mascot/cgi/peptide_view.pl?file=../data/20121122/F008183.dat&amp;query=71&amp;hit=1))
 **32 - 60 3168.4436 3167.4363 3167.6436 -0.2073 1 R.LLAEMWQGLGLAADAGELLFQAPERELAR.A** ([No match](http://iicbgps/mascot/cgi/peptide_view.pl?file=../data/20121122/F008183.dat&amp;query=75&amp;hit=1))
 **61 - 71 1243.5612 1242.5539 1242.6455 -0.0916 0 R.AAEEELLAELR.R** ([No match](http://iicbgps/mascot/cgi/peptide_view.pl?file=../data/20121122/F008183.dat&amp;query=13&amp;hit=1))
 **61 - 72 1399.6539 1398.6466 1398.7466 -0.1000 1 R.AAEEELLAELRR.M** ([Ions score 25](http://iicbgps/mascot/cgi/peptide_view.pl?file=../data/20121122/F008183.dat&amp;query=21&amp;hit=1))
 **61 - 72 1399.6539 1398.6466 1398.7466 -0.1000 1 R.AAEEELLAELRR.M** ([No match](http://iicbgps/mascot/cgi/peptide_view.pl?file=../data/20121122/F008183.dat&amp;query=22&amp;hit=1))
 **75 - 88 1528.6393 1527.6320 1527.7390 -0.1069 0 R.SSQPTQGEQGTRPR.R** ([No match](http://iicbgps/mascot/cgi/peptide_view.pl?file=../data/20121122/F008183.dat&amp;query=30&amp;hit=1))
 **75 - 88 1528.6393 1527.6320 1527.7390 -0.1069 0 R.SSQPTQGEQGTRPR.R** ([No match](http://iicbgps/mascot/cgi/peptide_view.pl?file=../data/20121122/F008183.dat&amp;query=31&amp;hit=1))
 **75 - 94 2266.9866 2265.9793 2266.1349 -0.1555 1 R.SSQPTQGEQGTRPRRPTPMR.G** ([No match](http://iicbgps/mascot/cgi/peptide_view.pl?file=../data/20121122/F008183.dat&amp;query=65&amp;hit=1))



>P1;F83432
regulator in type III secretion PA1705 [imported] - Pseudomonas aeruginosa (strain PAO1)
C;Species F83432: Pseudomonas aeruginosa
C;Species Q9I326_PSEAE: Pseudomonas aeruginosa.
C;Species AAG05094: Pseudomonas aeruginosa PAO1
C;Date: 15-Sep-2000 #sequence_revision 15-Sep-2000 #text_change 09-Jul-2004
C;Accession: F83432
R;Stover, C.K.; Pham, X.Q.; Erwin, A.L.; Mizoguchi, S.D.; Warrener, P.; Hickey, M.J.; Brinkman, F.S.L.; Hufnagle, W.O.; Kowalik, D.J.; Lagrou, M.; Garber, R.L.; Goltry, L.; Tolentino, E.; Westbrook-Wadman, S.; Yuan, Y.; Brody, L.L.; Coulter, S.N.; Folger, K.R.; Kas, A.; Larbig, K.; Lim, R.M.; Smith, K.A.; Spencer, D.H.; Wong, G.K.S.; Wu, Z.; Paulsen, I.T.; Reizer, J.; Saier, M.H.; Hancock, R.E.W.; Lory, S.; Olson, M.V.
Nature 406, 959-964, 2000
A;Title: Complete genome sequence of Pseudomonas aeruginosa PA01, an opportunistic pathogen.
A;Reference number: A82950; MUID:20437337; PMID:10984043
A;Accession: F83432
A;Status: preliminary
A;Molecule type: DNA
A;Residues: 1-98
A;Cross-references: UNIPROT:Q9I326; UNIPARC:UPI00000C5454; GB:AE004597; GB:AE004091; NID:g9947671; PIDN:AAG05094.1; GSPDB:GN00131; PASP:PA1705
A;Experimental source: strain PAO1
C;Genetics:
A;Gene: pcrG; PA1705
C;SRCDB PIR2
C;IDN_TREMBL Q9I326_PSEAE;
C;IDN_GENBANK AAG05094;

| **Mascot:**  <http://www.matrixscience.com/> |
| --- |
