# Supplementary material for: PcrG protects the two long helical oligomerization domains of PcrV, by an interaction mediated by the intramolecular coiled-coil region of PcrG
Source: BMC Struct Biol. 2014 Jan 24;14:5. doi: 10.1186/1472-6807-14-5 (PMC3904411; doi:10.1186/1472-6807-14-5)
Supplement: Additional file 15 — Ramachandran Plot for the model of ∆PcrG (13–72) . For Validation of the model of ∆PcrG(13–72), PROCHECK server was used, which generated the corresponding Ramachandran Plot showing residues in the most favoured, allowed and disallowed region in the model. [file 1472-6807-14-5-S15.pdf]

PROCHECK

Ramachandran Plot

DeltaPcrG

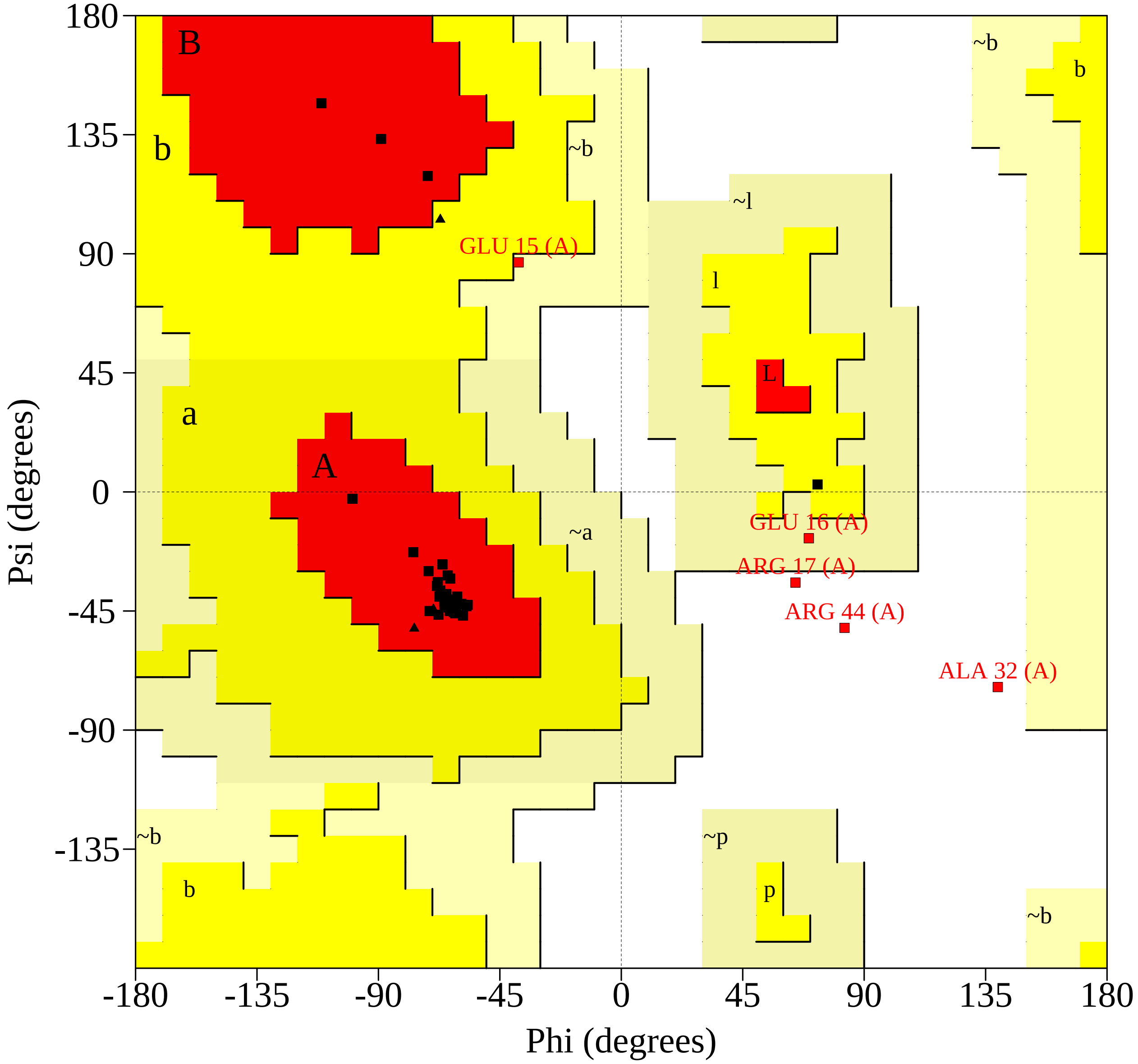

Plot statistics

Residues in most favoured regions [A,B,L]	47	88.7%
Residues in additional allowed regions [a,b,l,p]	1	1.9%
Residues in generously allowed regions [~a,~b,~l,~p]	2	3.8%
Residues in disallowed regions	3	5.7%
	----	------
Number of non-glycine and non-proline residues	53	100.0%
Number of end-residues (excl. Gly and Pro)	2	
Number of glycine residues (shown as triangles)	4	
Number of proline residues	1	

Total number of residues	60	

Based on an analysis of 118 structures of resolution of at least 2.0 Angstroms and R-factor no greater than 20%, a good quality model would be expected to have over 90% in the most favoured regions.

DeltaPcrG_01.ps
