# Supplementary material for: PcrG protects the two long helical oligomerization domains of PcrV, by an interaction mediated by the intramolecular coiled-coil region of PcrG
Source: BMC Struct Biol. 2014 Jan 24;14:5. doi: 10.1186/1472-6807-14-5 (PMC3904411; doi:10.1186/1472-6807-14-5)
Supplement: Additional file 17 — Ramachandran Plot for the model of ∆PcrG (13–72) -PcrV generated by molecular docking. For Validation of the model of ∆PcrG(13–72)-PcrV, PROCHECK server was used, which generated the corresponding Ramachandran Plot showing residues in the most favoured, allowed and disallowed region in the model. [file 1472-6807-14-5-S17.pdf]

PROCHECK

Ramachandran Plot

DeltaPcrGPcrV

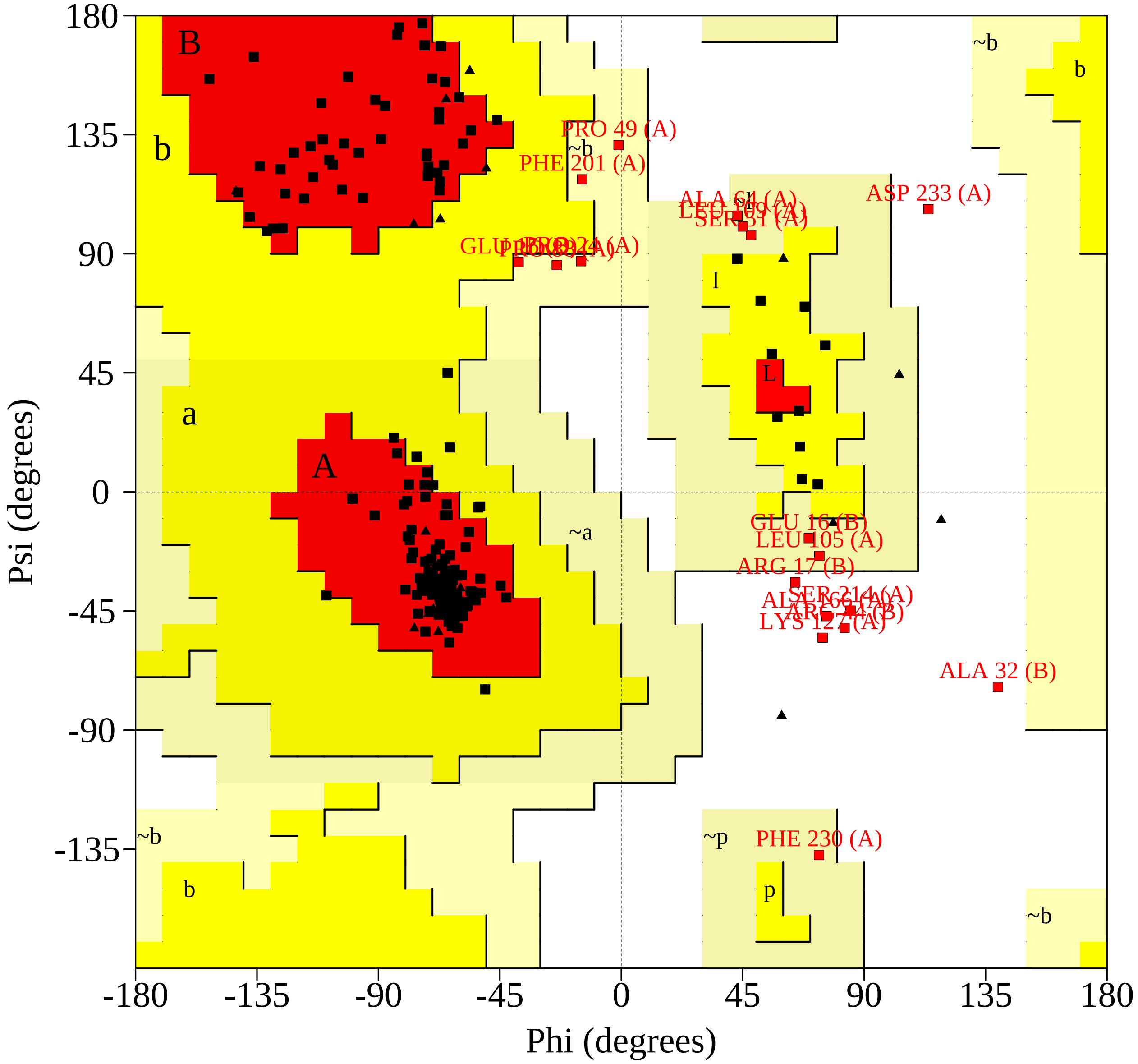

Plot statistics

Residues in most favoured regions [A,B,L]	281	89.2%
Residues in additional allowed regions [a,b,l,p]	19	6.0%
Residues in generously allowed regions [~a,~b,~l,~p]	8	2.5%
Residues in disallowed regions	7	2.2%
	----	------
Number of non-glycine and non-proline residues	315	100.0%
Number of end-residues (excl. Gly and Pro)	4	
Number of glycine residues (shown as triangles)	21	
Number of proline residues	14	

Total number of residues	354	

Based on an analysis of 118 structures of resolution of at least 2.0 Angstroms and R-factor no greater than 20%, a good quality model would be expected to have over 90% in the most favoured regions.

DeltaPcrGPcrV_01.ps
